# Polyaniline‐Coated MOFs Nanorod Arrays for Efficient Evaporation‐Driven Electricity Generation and Solar Steam Desalination

**DOI:** 10.1002/advs.202004552

**Published:** 2021-02-01

**Authors:** Zhuoyi Li, Xu Ma, Danke Chen, Xinyi Wan, Xiaobin Wang, Zhou Fang, Xinsheng Peng

**Affiliations:** ^1^ State Key Laboratory of Silicon Materials School of Materials Science and Engineering ERC of Membrane and Water Treatment Zhejiang University Hangzhou 310027 P. R. China

**Keywords:** desalination, hybrid metal‐organic framework membranes, solar‐heating interfacial evaporation, water evaporation‐driven electricity generation

## Abstract

Though evaporation‐driven electricity generation has emerged as a novel eco‐friendly energy and attracted intense interests, it is typically demonstrated in pure water or a very low salt concentration. Integrating evaporation‐driven electricity generation and solar steam desalination simultaneously should be more promising. Herein, a polyaniline coated metal‐organic frameworks (MOFs) nanorod arrays membrane is synthesized which inherits the merits of both polyaniline and MOFs, demonstrating nice stability, good interfacial solar steam desalination, and evaporation‐driven electricity generation. Moreover, an integrated system based on this hybrid membrane achieves good interfacial solar‐heating evaporation and prominently enhanced evaporation‐driven electricity generation under one sun. Notably, the realization of effective seawater desalination and efficient evaporation‐driven electricity generation simultaneously by the non‐carbon‐based materials is reported for the first time, which provides a new alternative way for cogenerating both freshwater and electricity by harvesting energy from seawater and solar light.

## Graphical abstract

The integration of interfacial solar‐heating evaporation and evaporation‐driven electricity generation is achieved based on a rationally designed hybrid membrane with polyaniline coating on the metal‐organic frameworks’ nanorod arrays. This membrane demonstrates effective sea water desalination and efficient evaporation‐driven electricity generation simultaneously under solar light.

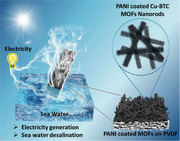

## Introduction

The unrestrained use of non‐renewable resources has caused intractable pollution to the environment and resulted in an intensified energy and resource crisis. The dwindling freshwater resources and lack of electricity are still the main problems hindering the development of some rural areas in the world. Many studies have been done about generating electricity^[^ ^1^, ^2^, ^3^ ^]^ and collecting freshwater^[^ ^4^, ^5^, ^6^ ^]^ from the ambient surroundings, but most of them are independent.

Recently, researches about some new kinds of electricity generation have arisen intense interests.^[^ ^7^, ^8^ ^]^ Such as moving droplets that could generate millivolts voltage on graphene due to the formation of waving potential.^[^ ^7^ ^]^ Different from the waving potential, a voltage of about 1 V has been achieved on carbon black originating from a streaming potential.^[^ ^8^ ^]^ Streaming potential is always generated with the help of an external driving force like salinity gradients or pressures that drive the ionic solution to pass through the charged microchannels on the solid surface. The natural process of the evaporation of water at the interface is a promising driving force to generate a streaming voltage. Thus, this evaporation‐driven electricity generation provides a new idea of generating electricity by making full use of the ubiquitous natural water evaporation process which harvests thermal energy from the ambient environment and turns the sensible energy into latent heat.

According to the traditional streaming potential theory,^[^ ^9^, ^10^, ^11^ ^]^ a distinct evaporation‐driven potential will be generated if satisfied are the following requirements: 1) good hydrophilic microchannels; 2) the size of the microchannels must meet the ion permeability and be close to or smaller than the Debye length; 3) the overlaps of the electric double layers (EDLs) form on the surface of microchannels. Thus, the material suitable for evaporation‐driven streaming potential should have an appropriate pore size for ion and water transportation and a high surface potential for the formation of overlapped EDLs.^[^ ^12^ ^]^

Carbon‐based materials such as graphene,^[^ ^13^, ^14^ ^]^ carbon black,^[^ ^8^, ^15^ ^]^ and carbon nanotubes,^[^ ^16^ ^]^ etc. have been wildly utilized for evaporation‐driven electricity. Though low‐cost carbon‐based materials show good electricity generation performance, the preparation process of those always involved a high temperature carbonization process^[^ ^17^ ^]^ which caused more energy consumption. Other materials like fabric^[^ ^18^ ^]^ and natural wood^[^ ^19^ ^]^ have also been proved to generate evaporation‐driven electricity but with a relatively low output power density.

Recently, due to the high porosity, charged surface, and hydrophilicity of metal‐organic frameworks (MOFs) based materials, MOFs have been considered to be a promising candidate for evaporation‐driven electricity generation such as in Ref. ^[^ ^12^ ^]^. However, most of MOFs are regarded as “water sensitive”, namely not stable in a water environment. The degradation of MOFs usually happens on their surface where they have many defects and barely functional groups.^[^ ^20^, ^21^ ^]^ Thus, knowing how to increase the stability of MOFs in a water environment is worthwhile.^[^ ^22^, ^23^, ^24^ ^]^

Similar to the natural water evaporation process, solar energy is universal and accessible everywhere. Recently, solar‐heating interfacial evaporation is emerging as a promising strategy to promote water evaporation and generate freshwater using solar energy with a high evaporation rate and a high solar‐heat conversion efficiency.^[^ ^6^, ^25^, ^26^, ^27^, ^28^, ^29^ ^]^ Polyaniline (PANI) is famous for its high conductivity, flexibility, chemical stability, and good light absorption property and has been regarded as a competitive candidate for solar‐heating interfacial evaporation.^[^ ^30^, ^31^ ^]^ Consequently, effectively integrating the MOFs with polymers by rationally designing their structure is a promising way to not only enhance the stability of MOFs but also possess the properties from both MOFs and polymers. The hybrid MOFs materials are thought to have both an evaporation‐driven electricity generation ability and a solar‐heating interfacial evaporation property which will cogenerate electricity and freshwater from sea water. It will alleviate the severe situation of lacking energy and fresh water. However, except for few carbon‐based materials,^[^ ^13^, ^15^, ^16^, ^32^, ^33^ ^]^ MOFs‐based materials have not been explored for this area.

Herein, a hybrid membrane with a copper‐BTC MOFs [Cu_2_(OH)(BTC)(H_2_O)]_n_·2H_2_O (CBA) nanorod arrays coated by PANI (CBAP) on a polyvinylidene fluoride (PVDF) substrate was prepared at room temperature. This hybrid membrane not only maintained a suitable size of micropores, high porosity, surface area, negatively charged surface, and good hydrophilicity from CBA but also inherited a good light absorption property and good photo‐thermal conversion efficient from the coated PANI. Besides, the coated PANI remarkably improves the stability of the CBA MOFs’ layer. Thus, these merits of the hybrid membrane guarantee its good water evaporation‐driven electricity generation and efficient solar‐heating interfacial evaporation. Significantly, the synergy of the solar‐heating interfacial evaporation and evaporation‐driven electricity generation combined with the pyroelectric effect of the PVDF substrate achieved a good interfacial evaporation performance and distinctively enhanced evaporation‐driven electricity generation simultaneously not only in pure water but also in salt solutions under solar light. Notably, this membrane‐based device constantly generated a voltage up to 709.3 mV (0.1 mm NaCl solution) and a maximum output power density which was 15.377 mW m^−2^ (in 0.6 m NaCl solution, similar to sea water). Additionally, a rational integration of solar‐heating sea water desalination and electricity generation under one sun illumination was demonstrated. The sustainable output electricity was large enough to power small electronic devices. Our hybrid membrane provided a new alternative way for generating freshwater and evaporation‐driven electricity concurrently by harvesting energy from ambient surroundings.

## Results and Discussion

**Figure 1:**
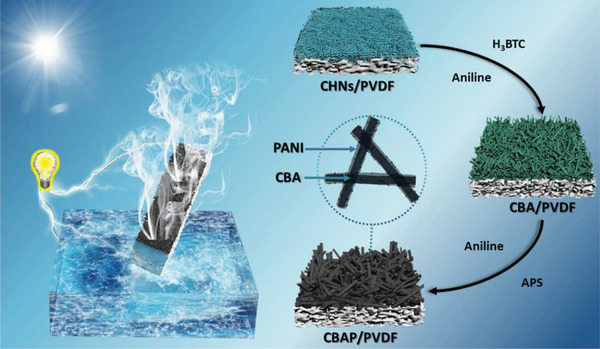
The synthetic process of CBAP membrane and the synergy of evaporation‐driven electricity generation and solar steam desalination.

**Figure 2:**
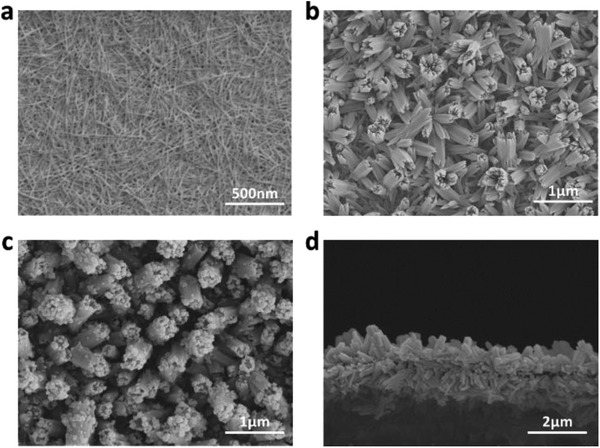
Surface SEM images of a) CHNs, b) CBA, and c) CBAP membrane. d) Cross‐section SEM images of CBAP membrane.

The synthesis process of the hybrid membrane CBAP membrane is illustrated in **Figure** **1**. Typically, three main steps were followed: 1) Blue copper hydroxide nanostrands (CHNs) membrane was obtained by filtering CHNs solution on a porous PVDF substrate (**Figure** **2**a; Figure S1a, Supporting Information); 2) the CHNs membrane was immersed in 1,3,5‐benzenetricarboxylic acid (H_3_BTC) and an aniline mixed solution for 2 h at room temperature to form a green CBA nanorod arrays membrane (Figure 2b; Figure S1b, Supporting Information); 3) the CBA membrane was transferred into a mixture of aniline ethanol solution and ammonium persulfate (APS) aqueous solution by polymerizing an aniline monomer on the surface of the CBA nanorod arrays at room temperature. Finally, a dark CBAP membrane (Figure S1c, Supporting Information) was prepared after 24 h with the CBA nanorod arrays coated by a thin‐layer of PANI.

The CBA phase was proved by the powder X‐ray diffraction (PXRD) pattern (Figure S2, Supporting Information) which matched well with the simulated pattern of [Cu_2_(OH)(BTC)(H_2_O)]*_n_*·2H_2_O.^[^ ^34^ ^]^ It reported that the solvent and the strength of base employed for the deprotonation of H_3_BTC played an essential role in the dimensionality and structure of the resulting framework in the synthesis process of Cu‐BTC complexes.^[^ ^35^ ^]^ In this work, we used water/ethanol (1:1) as a solvent and an aniline monomer as a morphology modulator where ethanol was a poor coordinating solvent, and aniline was a weak base for deprotonation, which lead to the rod‐like structure of CBA which enormously increased its surface area.

The powder XRD pattern of CBAP shows the same peaks as those of CBA (Figure S3, Supporting Information), implying the good structure maintenance of CBA in the polymerization process and it is also proved by the scanning electron microscope (SEM) images of the CBAP and CBA (Figure 2c,b) membrane with similar rod‐like structures. The cross‐section of the CBAP membrane shows a thickness of 2 µm (Figure 2d). However, the surface of the CBAP membrane with few particles seemed rougher than that of the CBA, indicating the formation of PANI on the surface of the CBA nanorod arrays. Similarly, the TEM images and element mapping of the CBAP (Figure S4, Supporting Information) also display that the surface of the rod‐like CBA was coated by PANI. The Fourier transform infrared (FTIR) spectrum of the CBAP (red line in Figure S5, Supporting Information) shows the absorption peaks at 802, 1288, 1209, 1024, 1489, and 1580 cm^−1^ corresponding to the PANI were observed, indicating the successful polymerization of the aniline.^[^ ^36^, ^37^ ^]^

The stability of the resulting membranes in water was first investigated. Figure S6, Supporting Information, shows the XRD patterns of the CBA membrane immersed in water for 3 days and the CBAP membrane immersed in sea water (600 mm NaCl solution) for 14 days, respectively. It is clear that the crystal structures of the CBA membrane had been destroyed indicating its instability in water. But the crystal structure of the CBAP membrane remained good, even in sea water. Thus, although the structure of the CBA was not stable in water its stability in water had been improved effectively after being coated by PANI. This is prerequisite for solar steam desalination as well as evaporation‐driven electricity generation.

**Figure 3:**
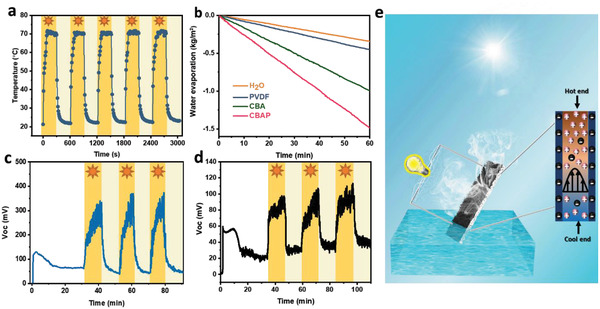
a) The temperature variation of CBAP membrane in dry state under 1 sun illumination. b) Mass change of water over time of CBA, CBAP, PVDF membranes, and without any absorbers under 1 sun illumination, respectively. Measured *V*_oc_ of c) CBAP membrane and d) PVDF membrane immersed in deionized water when the solar light (1 sun illumination) was periodically turned on and turned off. e) Mechanism of evaporation‐driven electricity generated by the CBAP membrane.

The surface color of the CBAP membrane got darker with the increasing polymerization time and the CBAP membrane polymerized for 24 h had an efficient and broadband absorption of about 94% from 250 to 2 500 nm (Figure S7, Supporting Information). The optical photographs and surface temperature of the membranes with the different polymerization time of PANI in the dry state under one sun illumination, which reflected their photothermal conversion ability,^[^ ^32^ ^]^ are shown in Figure S8, Supporting Information. The surface temperature of the CBAP membranes increased from 57.5 to 70.7 °C with a polymerization time from 2 to 24 h. The CBAP membrane with the PANI, polymerized for 24 h, exhibited a rapid temperature increase, which could reach 70.4 °C within 40 s (**Figure** **3**a). The PVDF, CBA, and CBAP membranes exhibited water contact angles of ≈58.5°, ≈0°, and ≈24.2° after 3 s (Figure S9, Supporting Information), respectively, indicating the super‐hydrophilicity of the CBA membrane and good hydrophilicity of the CBAP membrane.

The large surface area, porous structure, efficient light absorption, good hydrophilicity, and good photothermal conversion ability of CBAP membrane laid the solid foundations for its effective and efficient water evaporation performance, as measured by a homemade device (Figure S10, Supporting Information, more details in Experimental Section). The mass change over time under one sun illumination is shown in Figure 3b. The evaporation rates of PVDF, CBA, and CBAP membranes (obtained from the slope of the mass change curves and the projected areas of these membranes were equal to their evaporation areas) were 0.452, 1.001, and 1.442 kg m^−2^ h^−1^, respectively. During the evaporation process, the CBAP membrane was placed on a waterproof sponge floating on the water. The water was absorbed by a super‐hydrophilic tail made of paper and transported uphill by capillary force through the PVDF membrane to the CBAP membrane where the water transferred faster due to the super‐hydrophilicity of the CBA. The surface temperature of the water, PVDF, CBA, and CBAP membranes in the water‐wetted state after having been illuminated under one sun illumination for 60 min (Figure S11, Supporting Information) reached 25.1, 26.2, 33.6, and 38.4 °C, respectively. When illuminated under solar light, the PANI absorbed the solar energy and converted it into heat to evaporate water at the surface, resulting in a decrease of the surface temperature of membranes.^[^ ^38^ ^]^ The calculated solar‐vapor efficiency of PVDF, CBA, and CBAP membranes are 22.6%, 60.3%, and 90.8%, respectively (Table S1, Supporting Information). The low thermal conductivity (0.0927 W mK^−1^) of the CBAP membrane and the substrate also contributed to the good water evaporation rate and solar‐vapor efficiency of the CBAP membrane by reducing the heat conduction and convection dissipation from the heating surface to bulk water.

It has been shown that it is a promising way to convert the evaporation‐driven energy to electricity.^[^ ^8^ ^]^ The electricity generation performance of the CBAP membrane and PVDF substrate in deionized water without solar light was evaluated by a rationally designed device (Figure S12, Supporting Information, more details in the Experimental Section). The lower copper electrode was just immersed in deionized water, while the upper copper electrode was exposed to air (temperature was 25 °C, and relative humidity (RH) was 50%). The moment the CBAP membrane was in contact with the deionized water, no voltage was generated between the two electrodes. As the water was imbibed gradually by the membrane, a certain open‐circuit voltage (*V*_oc_) ≈47.4 mV and short‐circuit current (*I*_sc_) ≈0.06 µA was generated but only when the water diffused to the upper electrode, *V*_oc_ and *I*_sc_ between two electrodes was recorded and reached equilibrium after a certain time (Figure 3d; Figure S13a, Supporting Information). The existence of a large amount of negative charges on the membrane surface (Figure S14, Supporting Information) contributed a lot to the generation of *V*_oc_. When the membrane contacted with the aqueous solution, the absorbed solution passing through the porous network of the membrane formed an EDL due to the interaction between the ions in solution and the negatively charged surface of the channels. Thus the counter‐ions (such as H^+^) in the water flow were repelled and migrated uphill under a capillary driven force, resulting in the accumulation of the counter‐ions and creation of a charge polarization.^[^ ^19^, ^39^ ^]^ Consequently, a streaming potential was generated and reached equilibrium by the time all the pores were saturated (Figure 3e).

The results in Figure 3d and Figure S13b, Supporting Information, show that the PVDF membrane could also generate a *V*_oc_ of about 26.2 mV as expected. However, the *V*_oc_ generated by the PVDF is much lower than that of those generated by the CBAP membrane. Generally, streaming potential depends much on the flow velocity of the water and the transportation of ions, which rely on the wettability, the number of open nanopores, and the amount of surface charges of the membrane.^[^ ^40^ ^]^ The better the hydrophilicity and the greater the amount of open‐ended nanopores and surface charges, the easier the transportation of the water molecules and ions were. The surface zeta potential of pure PVDF, CBA membrane, and CBAP membrane was −31.4, −15.5, and −12.3 mV, respectively (Figure S14, Supporting Information). The increased surface potential of the CBAP membrane indicated the less negatively charged surface of the CBAP due to the coated PANI layer (with a positive zeta potential 19.0 mV). Usually, the surface potential of a material is related to its surface charge density.^[^ ^41^ ^]^ If the zeta potential is known, the surface charge density (*σ*) on the shear plane near the membrane surface can be calculated (see more details in Supporting Information), according to the Gouy–Chapman EDLs theory.^[^ ^42^, ^43^, ^44^ ^]^

Although the surface potential of the CBAP membrane was lower than that of the PVDF membrane, the rod‐like surface of the CBAP membrane was very rough compared to the smooth PVDF membrane surface, making the amount of surface charges of the CBAP membrane greater than that of the PVDF. Besides, the hydrophilicity of the PVDF membrane was not as good as that of the CBAP membrane, and the CBAP with a PVDF substrate showed more amounts of open‐ended nanopores than that of pure PVDF membrane. As a result, the *V*_oc_ generated by the pure PVDF membrane had little contribution to the *V*_oc_ generated by CBAP membrane when there was no solar light.

The electricity generation performance of CBAP membrane and PVDF substrate in deionized water under one sun illumination were further evaluated with a simulated solar light being turned on and turned off with 10 min intervals. The *V*_oc_ of CBAP membrane in pure water (Figure 3c) increased sharply and reached equilibrium at about 360.7 mV under 1 sun with an increase of 313.3 mV, about 6.6 times more than the *V*_oc_ without solar light (≈47.4 mV). But the *V*_oc_ rapidly decreased to the initial potential when the light turned off, showing a good light response. The corresponding *I*_sc_ light response was also recorded (Figure S14a, Supporting Information). *I*_sc_ was enhanced when the light was on while it returned to its original value when the light was off. Similarly, the *V*_oc_ generated by the PVDF (Figure 3d) was also increased (from 26.2 to 79.5 mV) under 1 sun illumination. This was much lower than that of CBAP membrane. The water evaporation performance of the CBAP membrane and the PVDF substrate during the electricity generation process were also evaluated in deionized water (Figure S15, Supporting Information). The evaporation rates of the PVDF and CBAP membranes (obtained from the slope of the mass change curves and calculated by using the projected area of the membrane which was smaller than its evaporation area in this 3D system) were 0.601 and1.900 kg m^−2^ h^−1^, respectively. The illumination of solar light enhanced the electricity generation performance by promoting the water evaporation rate since the *V*_oc_ was determined by the flow velocity of water inside the membrane, which was proportional to the water evaporation rate from the membrane surface. The PVDF membrane with a lower water evaporation rate showed less electricity generation under solar light. Apart from the different water evaporation performance of the CBAP membrane and the PVDF membrane, the different electricity generation performance under solar light was also attributed to the primary pyroelectric effect of the PVDF substrate.^[^ ^45^ ^]^ When illuminated under solar light, the PVDF generated pyroelectricity due to the temperature difference established between the hot solar irradiated surface and the underneath cold bulk water (Figure S16, Supporting Information). The surface temperature of the PVDF and the CBAP membrane in the water‐wetted state after being illuminated by one sun were 25.6 and 38.1 °C, respectively. The lower electrode immersed in water remained stable at about 25.0 °C. As a result, the enhanced water evaporation rate and the pyroelectricity generated by the PVDF membrane collaboratively contributed to the promoted electricity generation under solar light.

The influence of the electrolyte solution on electricity generation performance was further explored. As shown in Figures S17, S18, and S19, Supporting Information, the generated *V*_oc_ (Figure S19a, Supporting Information) and *I*_sc_ (Figure S19b, Supporting Information) in the NaCl solution without solar light were higher than those generated in deionized water because of the higher concentration of ions than that of deionized water (≈10^−7^ mol L^−1^). The *V*_oc_ increases sharply as the concentration increases from 0 to 0.1 mm, and further slowly increases when the concentration reaches 600 mm.

According to the traditional streaming potential theory,^[^ ^46^, ^47^ ^]^ the prerequisite of generating the streaming potential is the size of the microchannels must be close to or smaller than the Debye length. The Debye length in different NaCl concentrations were calculated and concluded in Table S2, Supporting Information. The Brunauer–Emmett–Teller (BET) surface area and pore size distribution results of CBAP membrane (Figure S20, Supporting Information) indicate its BET surface area is 250.77 m^2^ g^−1^ with pore size centered at 0.7 nm, similar to the those reported.^[^ ^48^ ^]^ 1D open channels with a size of 5 × 7 Å of the CBA crystal^[^ ^34^ ^]^ (Figure S21, Supporting Information) is far smaller than the Debye length in low concentrations of NaCl solution, but close to the Debye length in 600 mm NaCl solution. This might be the reason for the generation of *V*_oc_ in NaCl solution depends on concentration. The higher the concentration, the more cations are accumulated on the top leading to increased *V*_oc_. The *V*_oc_ variation under different concentrations under one sun (Figure S19a, Supporting Information) were different. The *V*_oc_ increased rapidly as soon as the light turned on in a low concentration range (0.1, 1, and 10 mm). Specifically, the maximum *V*_oc_ of 709.3 mV was achieved with an increase of 470.1 mV in 0.1 mm NaCl solution. This value was about 1.97 times larger than the *V*_oc_ (≈239.2 mV) without solar light. However, the *V*_oc_ decreased when the concentration was high (such as 600 mm) under solar light. The absorbed solar light enhanced the water evaporation rate and accelerated the transportation of ions. This was beneficial to the electricity generation when the concentration was low. However, when the electrolyte concentration was high, the saturated pores of CBAP would be partially blocked by the clogging of ions that hindered the transportation of water molecules leading to the drop of *V*_oc_ relatively. According to Raoult's law which means adding a non‐volatile solute to a volatile solvent will reduce the vapor pressure of solvent, thus reducing the volatility of solution, therefore, with the increase of NaCl concentration, the evaporation rate of the solution will decrease under the same conditions, which has a certain impact on the voltage. As for the low concentration (0.1 mm), the clogging of the pores was negligible compared with other higher concentrations. Correspondingly, it had the highest water evaporation rate under solar light. Thus, the solar light had a positive effect on the *V*_oc_ in the 0.1 mm NaCl solution, with the highest enhancement.

**Figure 4:**
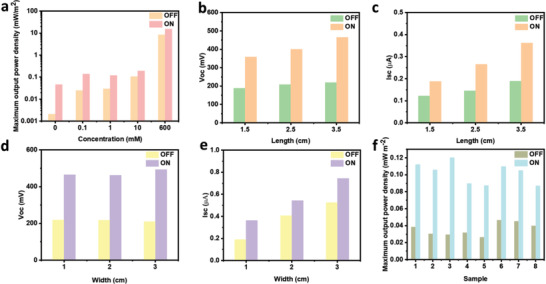
Maximum output power density a) *P*_max_ of CBAP membrane immersed in different concentrations of NaCl electrolyte solution. The b) *V*_oc_ and c) *I*_sc_ of CBAP membrane with different lengths and the same width of 1 cm. The d) *V*_oc_ and e) *I*_sc_ of CBAP membrane with different widths and the same length of 3.5 cm. f) Maximum output power density *P*_max_ of CBAP membranes. The CBAP membranes of (b–f) were immersed in 1 mm NaCl solution and the solar light (1 sun illumination) was periodically turned on and turned off.

Without solar light, both the *I*_sc_ and *V*_oc_ (Figure S19b, Supporting Information) increased with the NaCl's concentration. The impedances of CBAP membranes (Figure S22, Supporting Information) decreased with the NaCl concentration. However, under 1 sun, unlike the *V*_oc_ decreases in high concentration, the *I*_sc_ increased in all concentrations and reached a maximum of 34.715 µA in 600 mm NaCl solution. This can be explained by the results shown in Table S3, Supporting Information. On one hand, the *V*_oc_ increased but the impedance decreased in the low concentration range, making the *I*_sc_ increase. On the other hand, both the *V*_oc_ and the impedance decreased at high concentrations. But the decreased degree of the *V*_oc_ was less than that of the impedance's, so the *I*_sc_ still increased. The maximum output power density (*P*_max_) of the CBAP membrane in different concentrations are shown in **Figure** **4**a. It is clear that the *P*_max_ increased with the NaCl concentration and reached about 15.377 mW m^−2^ in the 600 mm NaCl solution though the *V*_oc_ slightly decreases under solar light.

The electricity generation performance of the CBAP membranes with different lengths (1.5, 2.5, and 3.5 cm) and width (1, 2, and 3 cm) were evaluated in the 1 mm NaCl solution, respectively. Having kept the width of 1 cm, it was found that both the *V*_oc_ (Figure 4b; Figure S23a,c, Supporting Information) and *I*_sc_ (Figure 4c; Figure S23b,d, Supporting Information) increased as the membrane length got longer due to the faster water evaporation rate caused by the increasing evaporation area. The longer the membrane was, the more the *V*_oc_ and *I*_sc_ increased. In the case of CBAP membranes with the same length of 3.5 cm but different widths (1, 2, and 3 cm), the *V*_oc_ (Figure 4d; Figure S24a,c, Supporting Information) showed no obvious change. But *I*_sc_ (Figure 4e; Figure S24b,d, Supporting Information) increased as the membrane width got wider. This may be attributed to more counterions having moved to the top electrode. When under solar light, the variations of the *V*_oc_ (Figure 4d) and *I*_sc_ (Figure 4e) of different widths were similar to the variations of the *V*_oc_ and *I*_sc_ of different lengths. Moreover, the influence of the angle between the membrane and horizontal plane was explored. The performance of membranes with different angles is shown in Figure S25, Supporting Information. Without solar light, the generated the *V*_oc_ decreased with the angle because the capillary force was counteracted by the gravity of ions and water molecules as the angle got larger. As for the *I*_sc_, there was no obvious difference. Under solar light, the variation of the *V*_oc_ with all angles increased with different variations. The smaller the angles, the more *V*_oc_ increased. This can be explained as follow (Figure S26, Supporting Information): As the angle increased, since the evaporation area of membrane is the same but the projected area was different, the larger the angle the smaller the projected area of membrane was. This results in less received solar energy because the solar intensity at the top of the membrane is 1 kW m^−2^. The less received solar energy caused a lesser increase of the *V*_oc_. The corresponding *I*_sc_ also increased under solar light but with no obvious difference for different angles. The overall generated electricity power and maximum output power density were concluded in Table S4, Supporting Information. The overall generated electricity power increased with the membrane area and decreased with the angle. However, the maximum output power densities (mW m^−2^) of these samples (Figure 4f) were very close. This indicates the CBAP membrane had uniform areal output power density. It makes it possible to enlarge the size of the membrane to get a higher output power.

In addition, the *V*_oc_ of the CBAP membrane in different electrolyte solutions (Figure S27, Supporting Information) were investigated. The *V*_oc_ of them varied in a small range and close to that of the NaCl. No obvious trends can be draw from these data. More detailed and systematic investigations are requested to deeply understand the influence of different electrolytes. Since the performance of NaCl was as the most stable system as well as it very close to sea water, the NaCl solution was chosen in this work.

The *V*_oc_ variation under different solar light intensities was studied (Figure S28a, Supporting Information). It was found that the *V*_oc_ increased as the light intensity increased from a 1 to 2 sun intensity. The higher light intensity further accelerated the evaporation rate and increased the temperature differences between the hot and the cold end of PVDF membrane when the concentration was low. Figure S28b, Supporting Information, shows the good stability and sustainability of the electricity generation of the CBAP membrane under the solar light.

**Figure 5:**
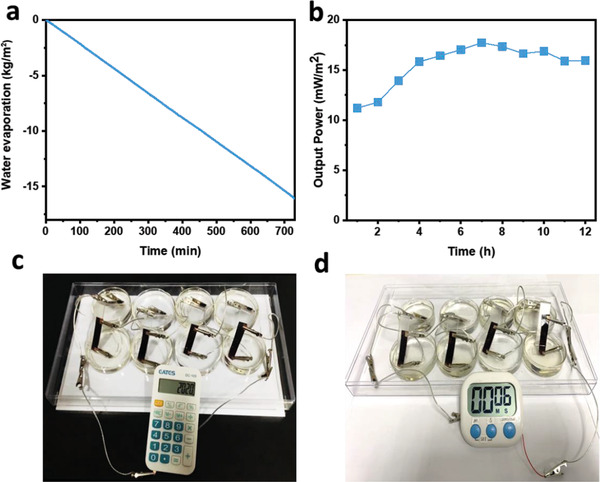
a) Mass change of sea water (600 mm NaCl) over time with CBAP as absorber during an electricity generation process. b) The sustainability of maximum output power density *P*_max_ of CBAP membrane in seawater (600 mm NaCl) desalination process under 1 sun and the solar light was periodically turned on and turned off. The scale‐up membrane based system can directly power c) a calculator and d) a timer.

Given is the good electricity generation performance of the CBAP membrane under solar light in the 600 mm NaCl solution: The water evaporation performance of the CBAP membrane was further evaluated in the 600 mm NaCl solution as simulated sea water. Considering the potential electrochemical corrosion of a copper electrode in sea water, we used platinum (Pt) strips as the electrodes when the electrolyte solution of the test was the 600 mm NaCl solution. As shown in **Figure** **5**a, the performance of sea water desalination during the electricity generation process can be sustained for over 12 h and the water evaporation rate was 1.866 kg m^−2^ h^−1^. *T* was slightly lower than that of the deionized water due to the increase of liquid‐vapor phase‐change enthalpy. The *V*_oc_ (Figure S29a, Supporting Information) declined a little at first and gradually increased with time. The reason was that at the beginning of illumination, the temperature difference between the top and bottom of PVDF was small. Therefore, the evaporation‐driven potential contributed majorly to the *V*_oc_. The evaporation‐driven potential decreased due to the partial blockage of nanopores caused by the clogging of ions. From time elongation, the temperature difference between the top and bottom of PVDF gradually increased. Thus, the pyroelectricity generated by PVDF via pyroelectric effect rose up and contributed to the later increment of *V*_oc_. Differently, the *I*_sc_ (Figure S29b, Supporting Information) and *P*_max_ (Figure 5b) in the desalination process were steady and sustainable. After continuously being operated for 12 h, the slightly formed salt crystals near to the top electrode (Figure S30a, Supporting Information) would disappear after turning off the solar light for 30 min (Figure S30b, Supporting Information). As a result, the CBAP membrane showed enormous potential in seawater desalination and evaporation‐driven electricity generation through the day over the night. The corresponding water evaporation rate and generated maximum output power density were competitive, even superior, to that of most reported materials (Table S5, Supporting Information).^[^ ^3^, ^8^, ^12^, ^13^, ^14^, ^15^, ^16^, ^18^, ^19^, ^39^ ^]^ To the best of our knowledge, this is the first report on a non‐carbon based material for seawater desalination and evaporation‐driven electricity generation simultaneously.

To further verify the feasibility and practicability of this device, we scaled up the device performance by connecting the eight CBAP membranes (3.5 × 1 cm^2^) in a series configuration. The output power was high enough to power small electronic devices like a calculator and timer (Figure 5c,d; Movies S1 and S2, Supporting Information) as well as to illume blue light‐emitting diodes (LED) (Movie S3, Supporting Information).

## Conclusion

In summary, a hybrid membrane composed of Cu‐BTC complex nanorod arrays coated with the PANI formed on the porous PVDF substrate was successfully fabricated. The Cu‐BTC nanorod arrays showed super hydrophilicity, plenty of 1D subnanochannels, and a large evaporation surface area. The cladding of the PANI endued the hybrid membrane with a good solar thermal conversion property and improved the stability of the Cu‐BTC nanorod arrays in water. These ensured the good solar‐heating interfacial evaporation performance with a solar‐vapor efficiency of 90.8% under a 1 sun illumination. In addition, the negatively charged surface of the porous hybrid membrane generated considerable electricity driven by the water evaporation. It could be further enhanced distinctively by illumination under solar light due to the improved water evaporation rate and the primary thermoelectric effect of the PVDF membrane. The *V*_oc_ and *I*_sc_ generated by the CBAP membrane under different electrolyte concentrations show a good light‐response property; both of them increased in low concentrations. The maximum open circuit voltage is as high as 709.3 mV, under 1 sun in the 0.1 mm NaCl. The maximum *I*_sc_ and output power density (34.715 µA and 15.377 mW m^−2^) was obtained in the 600 mm NaCl. Therefore, sea water desalination and electricity generation were simultaneously achieved by the CBAP membrane with a water evaporation of 1.866 kg m^−2^ h^−1^ and sustainable for over 12 h under 1 sun. A scale‐up device constructed in a series configuration was able to light up LEDs and power a calculator and a timer. The CBAP membrane can effectively and efficiently harvest the energy from the ubiquitous solar energy and natural evaporation process and generated freshwater and electricity concurrently. This provides a new avenue for solving the problem of scarce freshwater resources and energy problems at the same time.

## Experimental Section

##### Materials and Chemicals

Copper nitrate (Cu(NO_3_)_2_•3H_2_O), absolute ethanol, sodium chloride (NaCl), APS, and (NH_4_)_2_S_2_O_8_ were purchased from Sinopharm Chemical Reagent Co. Ltd. 2‐aminoethanol (NH_2_‐CH_2_CH_2_OH), 1,3,5‐benzenetricarboxylic acid (trimesic acid, H_3_BTC), and aniline (>98%) were purchased from Sigma‐Aldrich. PVDF membrane with a pore size ca. 220 nm and porosity of 70% were purchased from Whatman International Ltd. Ultrapure water of 18.2 MΩ produced by a Millipore Direct‐Q system was used throughout the experiments.

##### Synthesis of Copper Hydroxide Nanostrands (CHNs) Solution

The CHNs solution was prepared by mixing equal volume 4 mm copper nitrate (Cu(NO_3_)_2_) aqueous solution with 1.6 mm 2‐aminoethanol aqueous solution followed by aging for 24 h at room temperature.

##### Synthesis of CBA Membrane

Typically, 200 ml CHNs solution was filtered on a porous PVDF substrate to get a CHNs membrane and dried at room temperature. The blue CHNs membrane was then immersed in 5 mm H_3_BTC solution (water and ethanol were the solvents and aniline was an additive with their volume ratio of 50:50:1) and reacted for 2 h to get the green CBA membrane.

##### Synthesis of CBAP Membrane

First, 5 ml aniline was dissolved in 100 ml ethanol. Then, was added 5 ml 10mg ml^−1^ APS aqueous solution into the aniline ethanol solution and stirred for 5 min. Afterward, was transferred the CBA membrane to the mixture solution and reacted for 24 h. Finally, the dark CBAP membrane was obtained after being washed by ultrapure water and ethanol for three times, respectively.

##### Characterizations

The morphologies were characterized by scanning electronic microscopy (SEM) (Hitachi SU‐70 and SU 8010) and transmission electron microscope (TEM) (Hitachi HT‐7700) equipped with an X‐ray energy dispersive analysis (Oxford). The phases were characterized by powder XRD at 0.02° per step at room temperature using an XRD‐7000 (Shimadzu) instrument with Cu K*α* radiation. The FTIR spectroscopy was recorded on FT‐IR TENSOR 27 equipment using a KBr pellet in the range of 400–4000 cm^−1^. The surface zeta potential of membranes was measured in 0.1mm KCl electrolyte solution by Anton Paar, SurPASS3. The light absorptions of all samples were measured by using the ultraviolet‐visible‐near‐infrared spectrophotometer equipped with an integrating sphere (UV‐2600). And the contact angle was measured by a OCA 20 (Dataphysics) Contact angle meter. A FLIR E5‐xt infrared camera was used to take infrared photographs. A xenon light source (Perfectlight Solar Light Simulator; 1 kW m^−2^, 1 sun illumination) with an air mass 1.5 global (AM 1.5G) filter (UQG Optics). The RH and temperature were recorded using a humidity and temperature meter (MINGLE 101B). The N_2_ sorption analysis was conducted with a Micromeritrics specific area analyzer (Micromeritics, 3Flex, SN#340) at 77 K.

##### Surface Charge Density Calculation

The surface charge density (*σ*) on the shear plane near the membrane surface can be calculated by Equation (1).

(1) $$\begin{array}{cc} \sigma^{d}= & \left(Sign\;\zeta \;\right)\;{\left(2cERT\right)}^{\frac{1}{2}}\\ & \times {\left[v_{+}\exp\left(-\frac{z_{+}F\zeta }{RT}\right)+v_{-}\exp\left(-\frac{z_{-}F\zeta }{RT}\right)-v_{+}-v_{-}\right]}^{\frac{1}{2}} \end{array}$$

For 1:1 electrolyte solution, there are

(2) $$\sigma^{d}=-\varepsilon \kappa \zeta \sin\left(\frac{hF\zeta }{2RT}\right)/\left(\frac{F\zeta }{2RT}\right),\;\kappa^{-1}={\left[\frac{\varepsilon RT}{2F^{2}c}\;\right]}^{1/2}\;$$

When the potential is low (*ζ* ≤ 50*mV*), it is simplified to

(3) $$\sigma^{d}=\varepsilon \zeta /\kappa^{-1}$$

Where *c* is the molar concentration of the electrolyte solution (mol L^−1^), *ε* is the dielectric constant of the electrolyte solution, which is equal to *ε*_r_ × *ε*_0_, *v*_+_ and *v*_−_ are the stoichiometric coefficients of positive and negative ions that make up the electrolyte, and *z_+_* and *z_−_* are the valence states of positive and negative ions. Sign *ζ* represents the positive or negative sign of the zeta potential, *R* and *F* are gas constant (8.314 J mol^−1^ K^−1^) and Faraday constant (96487 C mol^−1^), *T* is the temperature (K), and *κ^−1^* is Debye length.

##### Electricity Generation Measurements

First, a setup was made by a sponge with an adjustable angle between its oblique plane and the horizontal plane. Then a smooth glass was used as a hard substrate and little claps to fix the electrodes (copper electrodes for low concentration test, Pt electrodes for seawater long‐term test) onto the bottom and the top of the CBAP membrane. The membrane with the glass substrate was attached onto the oblique plane of the sponge support. Besides, the bottom electrode has a long tail and connected with the probe through a long conductive cable. One end of this long conductive cable was connected to the tail of electrode and another end was fixed on the top of the balance. The positive electrode of a multimeter was connected to the top electrode of the CBAP membrane based device and the negative electrode of the multimeter was connected to the long tail of the bottom electrode. The simulated solar light was irradiated onto the membrane vertically from the top. Usually, the angle between the oblique plane of the sponge substrate and the horizontal plane was fixed at 45^o^ and the distance between two electrodes was 3.5 cm. When exploring the influence of the size and angle on performance, the angle and electrode distance would change accordingly. The *V*_oc_ and *I*_sc_ were measured by an Agilent 34401A Multimeter. The alternating current (A.C.) impedance was recorded by an electrochemical workstation (Vertex V01336).The maximum area output power density was calculated according to Equation (4):

(4) $$P_{\max}=\frac{1}{4}\times V_{\mathrm{oc}}\times I_{\mathrm{sc}}$$

##### Water Evaporation Measurements

The water evaporation experiments were performed on a homemade system (Figure S8, Supporting Information) with a xenon light source (Perfectlight Solar Light Simulator; 1 kW m^−2^, 1 sun illumination) with an air mass 1.5 global (AM 1.5G) filter (UQG Optics). The waterproof and insulated sponge was floating on the water with a small hole in its center. The membrane was placed on the sponge. The water was absorbed and transferred to the membrane by a superhydrophilic paper through the hole of the sponge, ensuring the sufficient water supply. The mass change of water was measured by an analytical balance (Sartorius, BSA124S), which real‐time communicated to a desktop computer for the evaluation of the evaporation rate and solar‐thermal conversion efficiency. In the experiment, the sea water was simulated by a 0.6 m NaCl aqueous solution. The water evaporation rate was calculated based on the projected area of the membranes. For an in‐plane water evaporation test, it was 3.14 cm^2^. For a water evaporation test in an electricity generation process, the projected area was 2.475 cm^2^.

##### Solar‐to‐Steam Efficiency Calculation

The formula $\eta \;=\frac{mh_{\mathrm{LV}}}{P_{\mathrm{in}}}\;$ is used for calculating the solar‐to‐steam efficiency (*η*),^[^ ^6^, ^25^ ^]^ where *m* is the water evaporation rate that removes the evaporation rate under dark conditions (0.12 kg m^−2^ h^−1^), *h*_LV_ is the total liquid‐vapor phase‐change enthalpy that contains sensible heat (*H*_sen_) and latent heat of evaporation (*∆H*_vap_), and *P*_in_ is the received power density of the solar irradiation on the absorber surface. It should be noted that the sensible heat and latent heat of water are all related to temperature. The *H*_sen_ can be calculated by *H*_sen_ *=* C × (*T*−*T*_0_), where C is specific heat of water (4.18 J g^−1^ K^−1^), *T*_0_ is the initial temperature of water, and *T* is the surface temperature of the membrane obtained from the IR camera (more details in Table S1, Supporting Information).

## Conflict of Interest

The authors declare no conflict of interest.

## Supporting information

Supporting Information

Supplemental Movie 1

Supplemental Movie 2

Supplemental Movie 3
