# Supplementary material for: Polyaniline‐Coated MOFs Nanorod Arrays for Efficient Evaporation‐Driven Electricity Generation and Solar Steam Desalination
Source: Adv Sci (Weinh). 2021 Feb 1;8(7):2004552. doi: 10.1002/advs.202004552 (PMC8025007; doi:10.1002/advs.202004552)
Supplement: Supplementary file 1 — Supporting Information [file ADVS-8-2004552-s001.pdf]

WILEY-VCH

© 2020 Wiley-VCH GmbH

ADVANCED SCIENCE
Open Access

Supporting Information

for *Adv. Sci.*, DOI: 10.1002/advs.202004552

Polyaniline Coated MOFs Nanorod Arrays for Efficient Evaporation-Driven Electricity Generation and Solar Steam Desalination

*Zhuoyi Li, Xu Ma, Danke Chen, Xinyi Wan, Xiaobin Wang, Zhou Fang, Xinsheng Peng**

Supporting Information

Polyaniline Coated MOFs Nanorod Arrays for Efficient Evaporation-Driven Electricity Generation and Solar Steam Desalination

*Zhuoyi Li, Xu Ma, Danke Chen, Xinyi Wan, Xiaobin Wang, Zhou Fang, Xinsheng Peng**

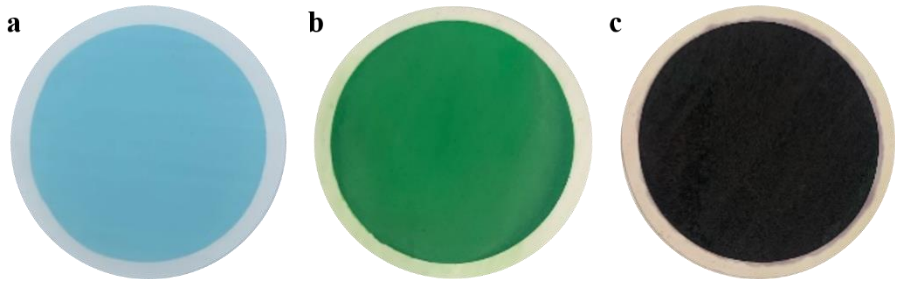

Figure S1. The optical photographs of CHNs (a), CBA (b) and CBAP (c) membrane.

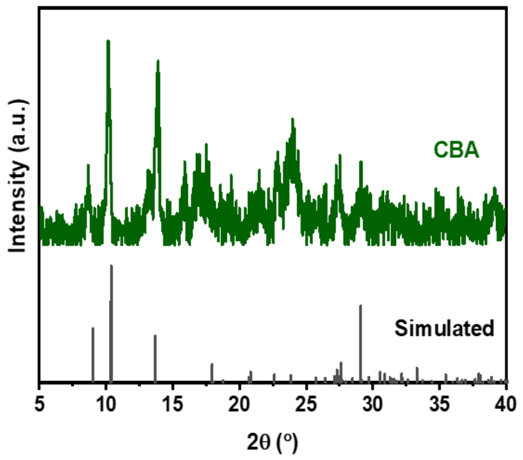

Figure S2. The XRD pattern of CBA membrane and simulated XRD pattern of $[Cu_2(OH)(BTC)(H_2O)]_n{\cdot}2nH_2O$.

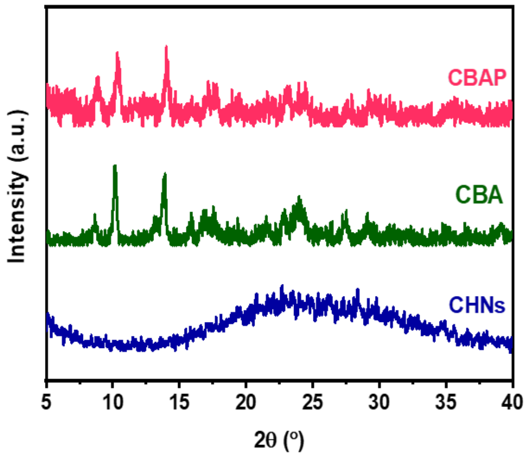

Figure S3. The powder XRD patterns of CHNs, CBA and CBAP.

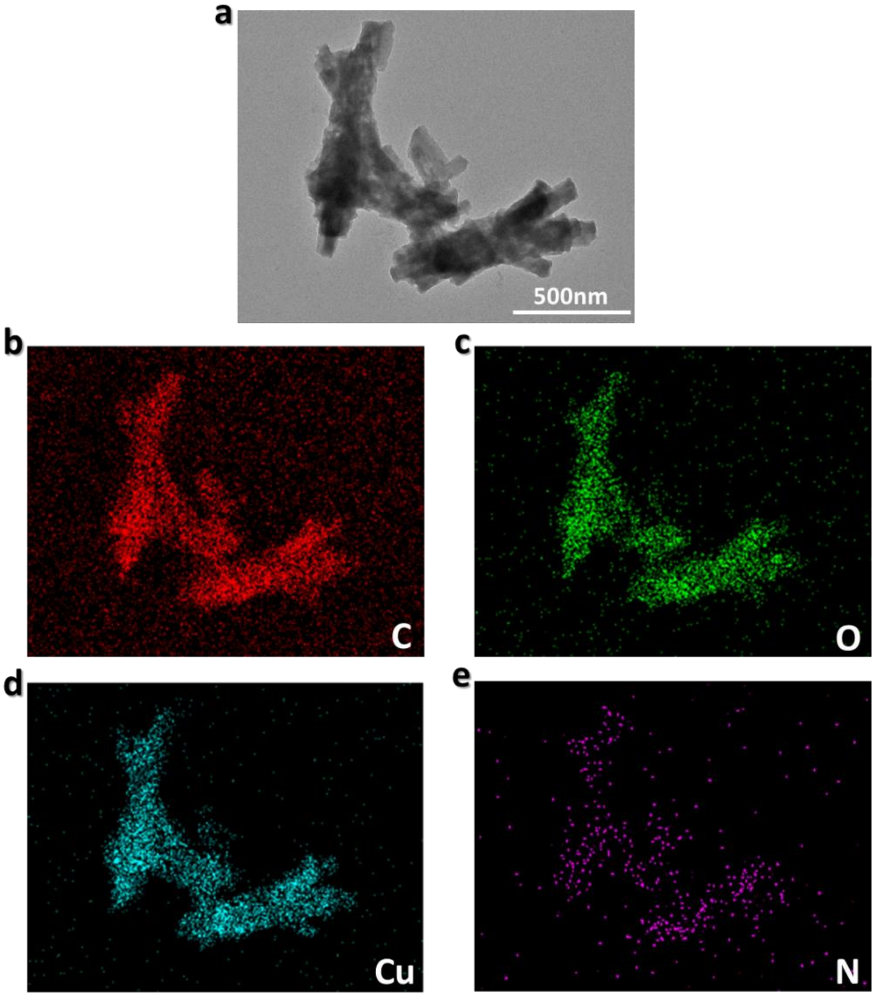

Figure S4. TEM image of CBAP (a) and EDS mapping result of Carbon (b), Oxygen (c), Copper (d) and Nitrogen (e) in CBAP membrane.

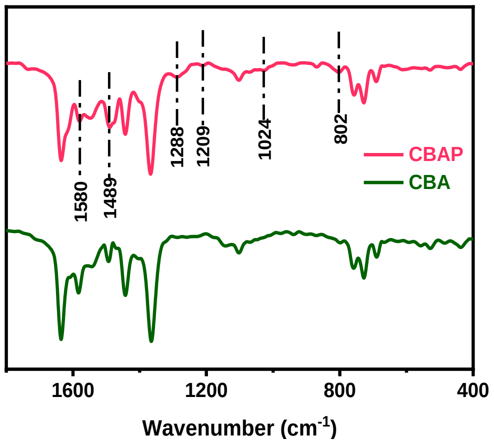

Figure S5. The FTIR spectra of the CBA and CBAP membranes across 1800–400 cm^{-1}.

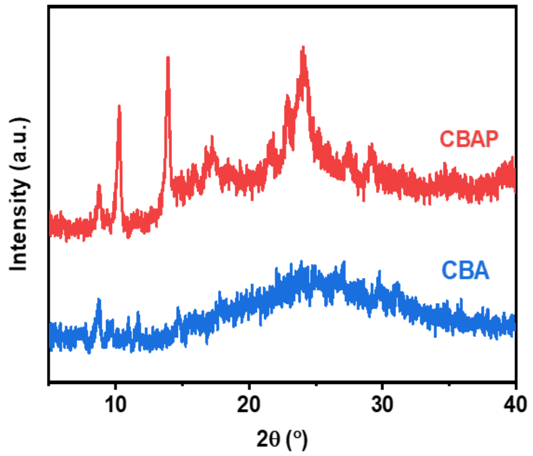

Figure S6. The powder XRD patterns of CBA membrane immersed in water for 3 days and CBAP membrane immersed in 600 mM NaCl solution for 14 days.

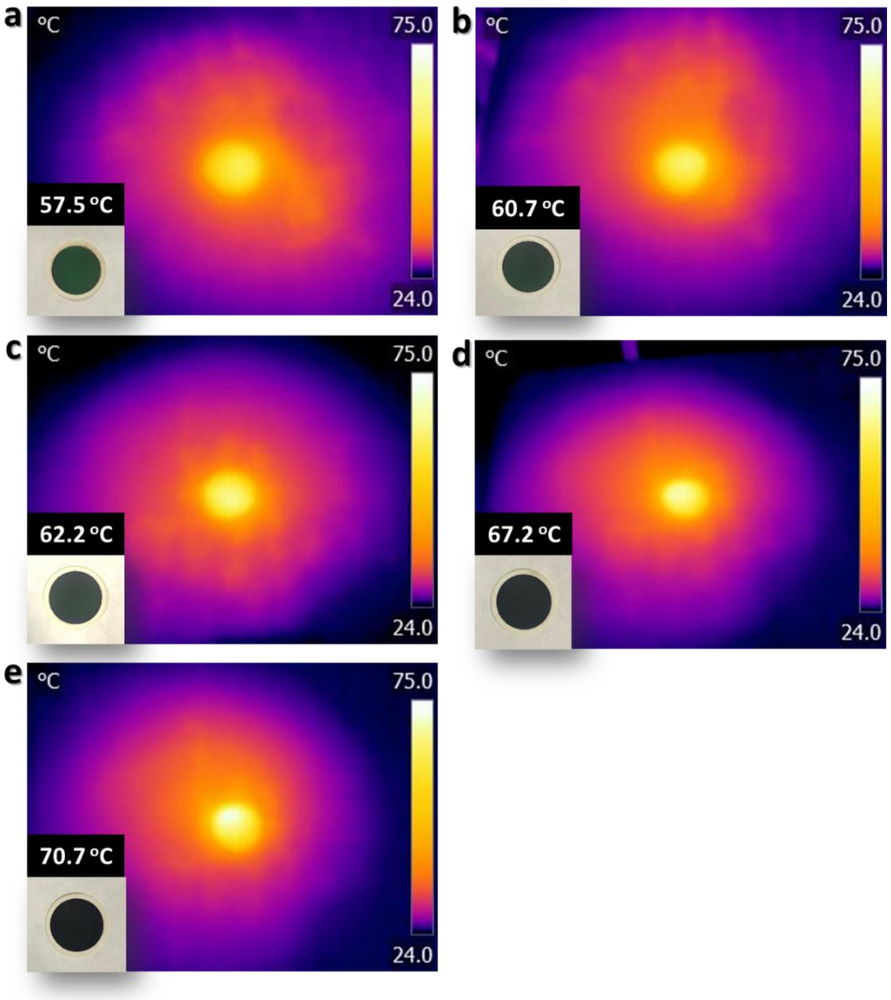

Figure S7. The surface temperature of the CBAP membranes with polymerization of PANI for 2h (a), 4h (b), 8h(c), 12h(d) and 24h (e), respectively, in dry state under 1 sun illumination. Inset is the optical photographs of the CBAP membranes.

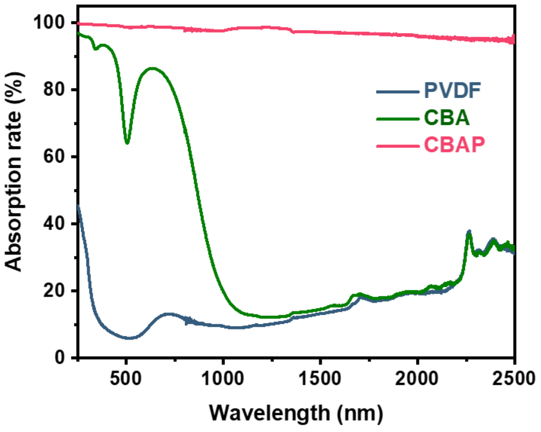

Figure S8. The light absorption spectra of the PVDF, CBA and CBAP membranes across 250–2500 nm.

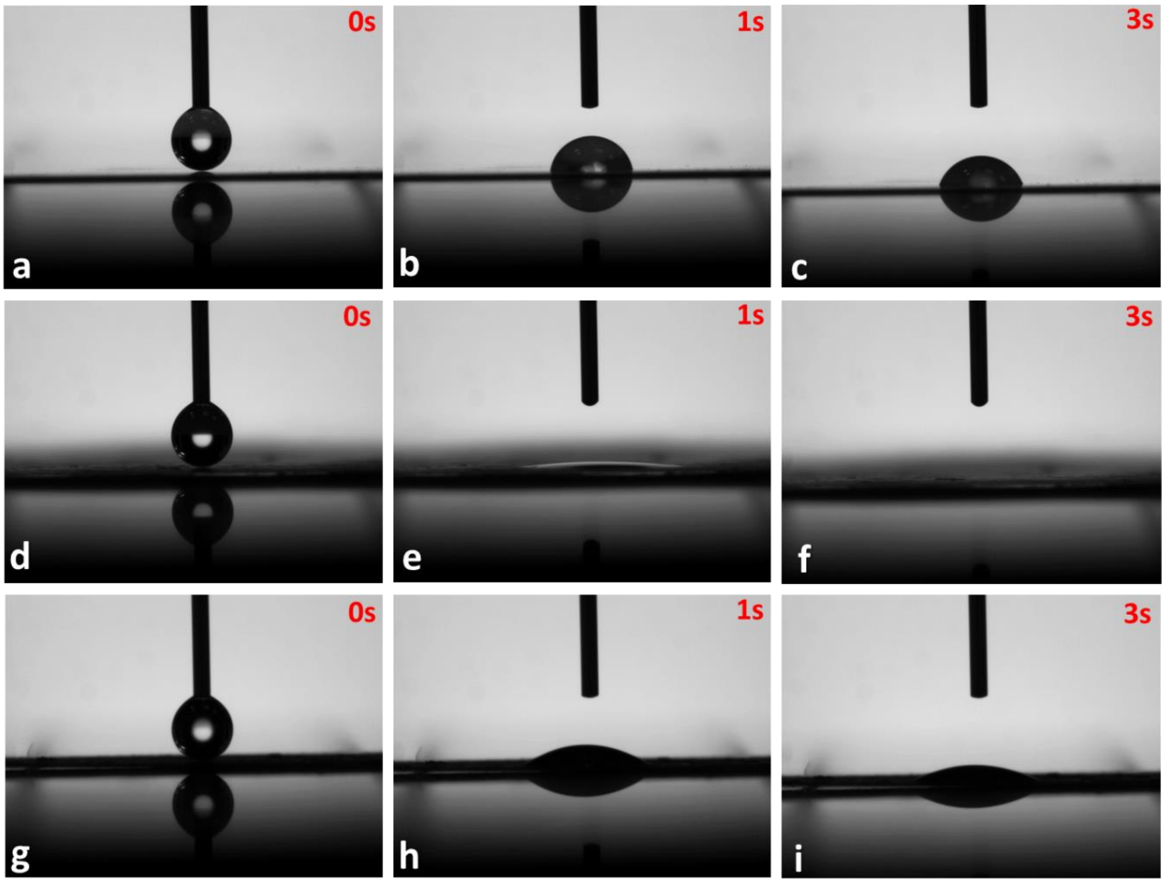

Figure S9. The water contact angle of PVDF (a, b and c), CBA (d, e and f), and CBAP (g, h and i) membranes in the different shooting time (0 s, 1 s and 3 s), respectively.

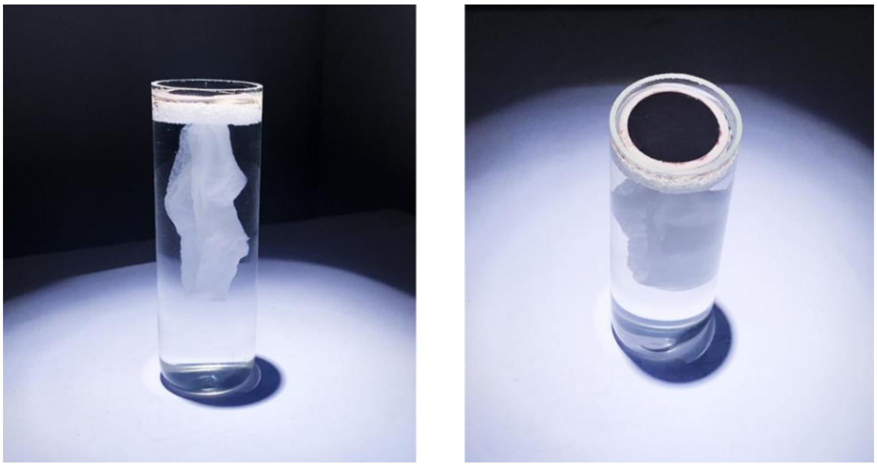

Figure S10. The optical photographs of the homemade system for water evaporation measurements.

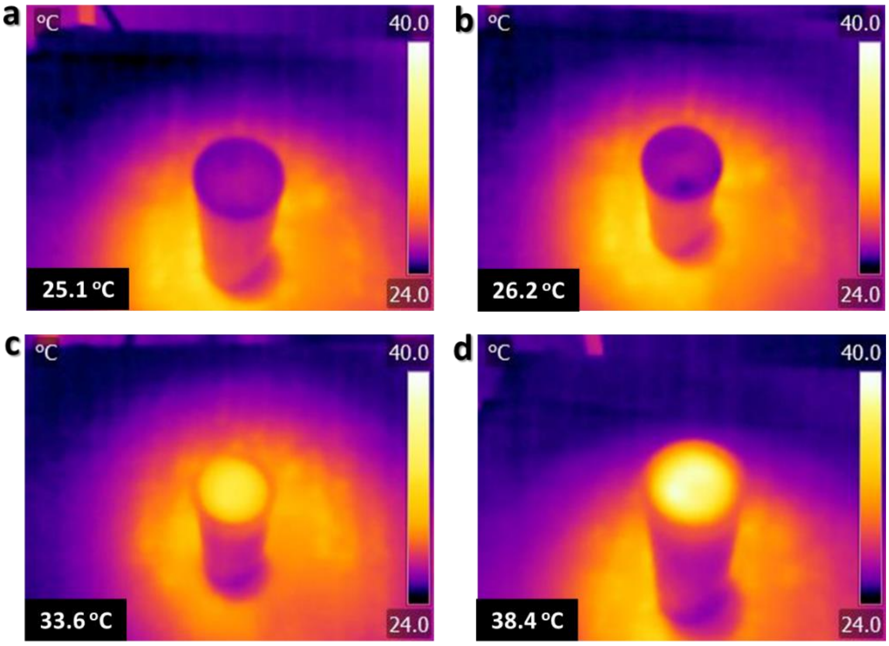

Figure S11. Infrared photographs of pure water (a), PVDF membrane (b), CBA membrane (c) and CBAP membrane (d) in water-wetted state under 1 sun illumination.

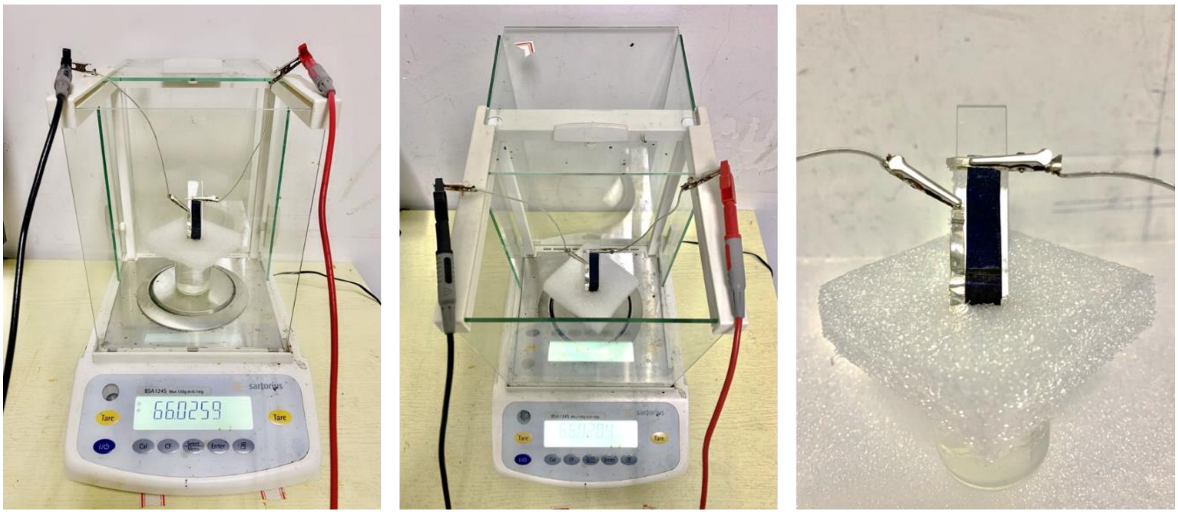

Figure S12. The optical photographs of the homemade system for electricity generation and solar water evaporation measurements.

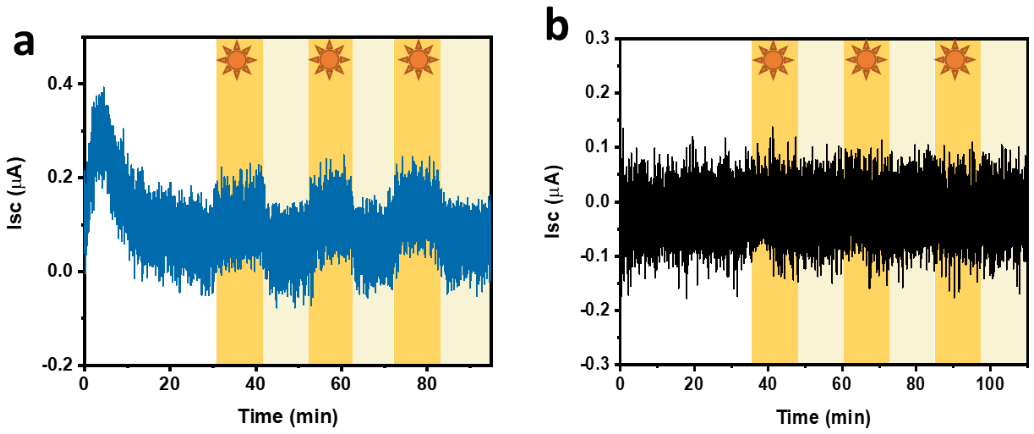

Figure S13. Measured I_{sc} of CBAP membrane (a) and PVDF membrane (b) under 1 sun illumination. The electrolyte solution is deionized water and the solar light was periodically turned on and turned off.

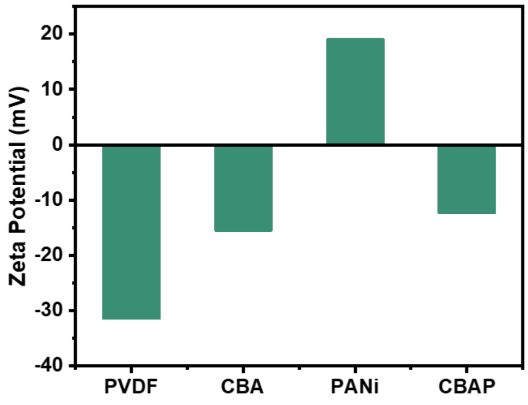

Figure S14. Surface potential of different membranes.

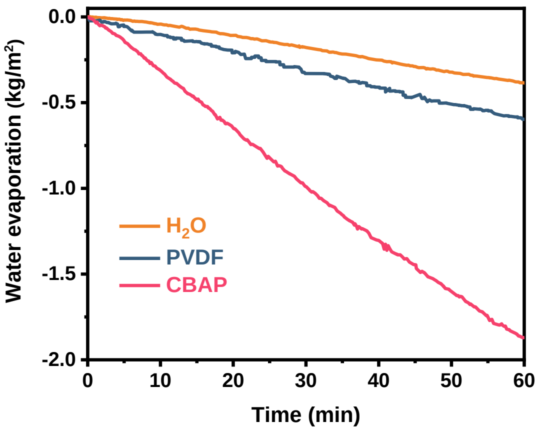

Figure S15. Mass change of water over time with CBAP, PVDF membrane and without any absorbers under 1 sun illumination in electricity generation process.

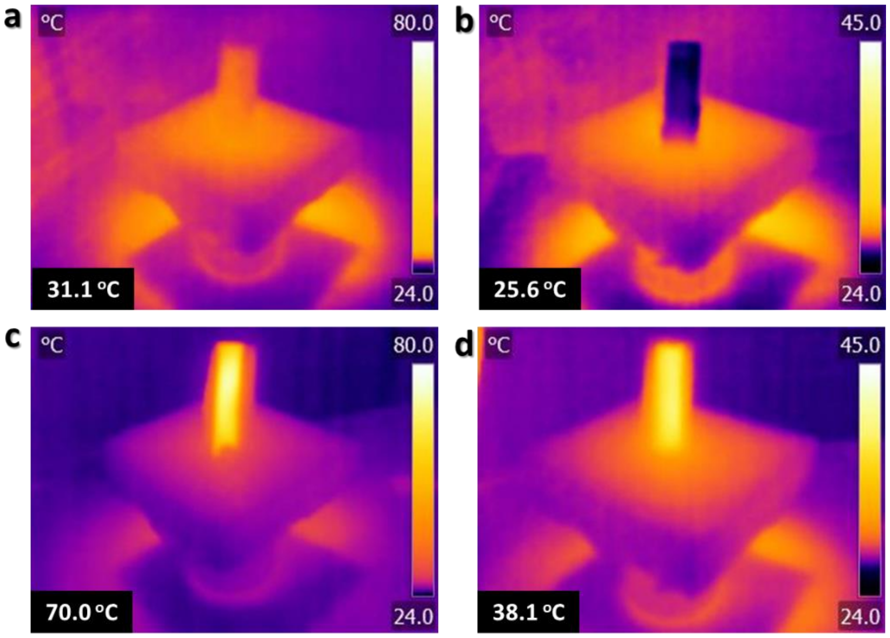

Figure S16. Infrared photographs of PVDF membrane (a) (b) and CBAP membrane (c)(d) in dry and water-wetted state under 1 sun illumination in the electricity generation process.

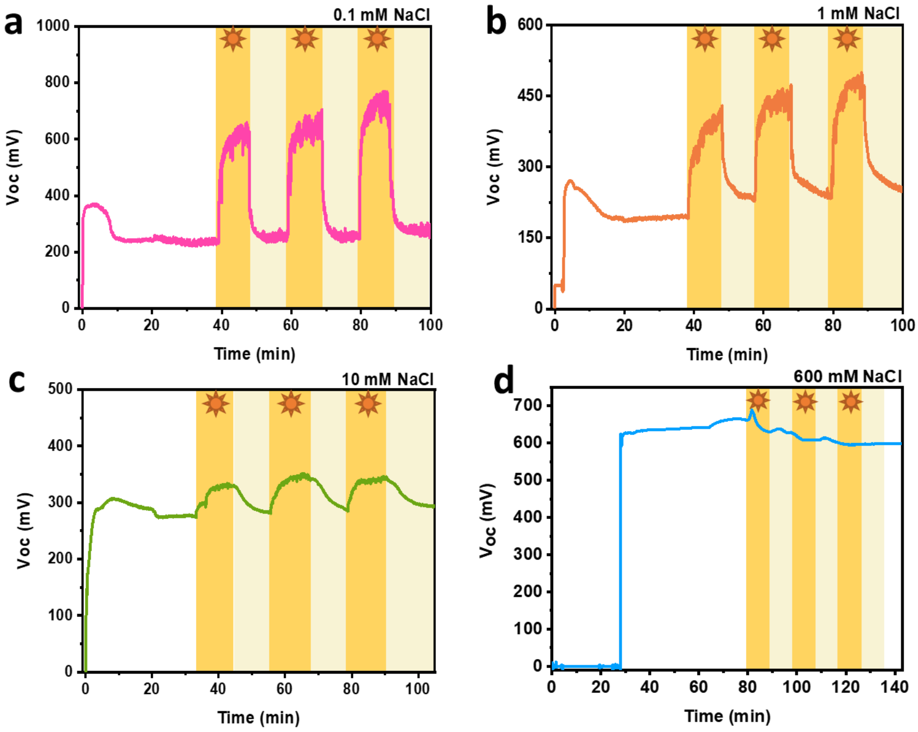

Figure S17. Measured V_{oc} of CBAP membrane immersed in 0.1 mM (a), 1 mM (b), 10 mM (c) and 600mM (d) NaCl electrolyte solution when the solar light (1 sun illumination) was periodically turned on and turned off.

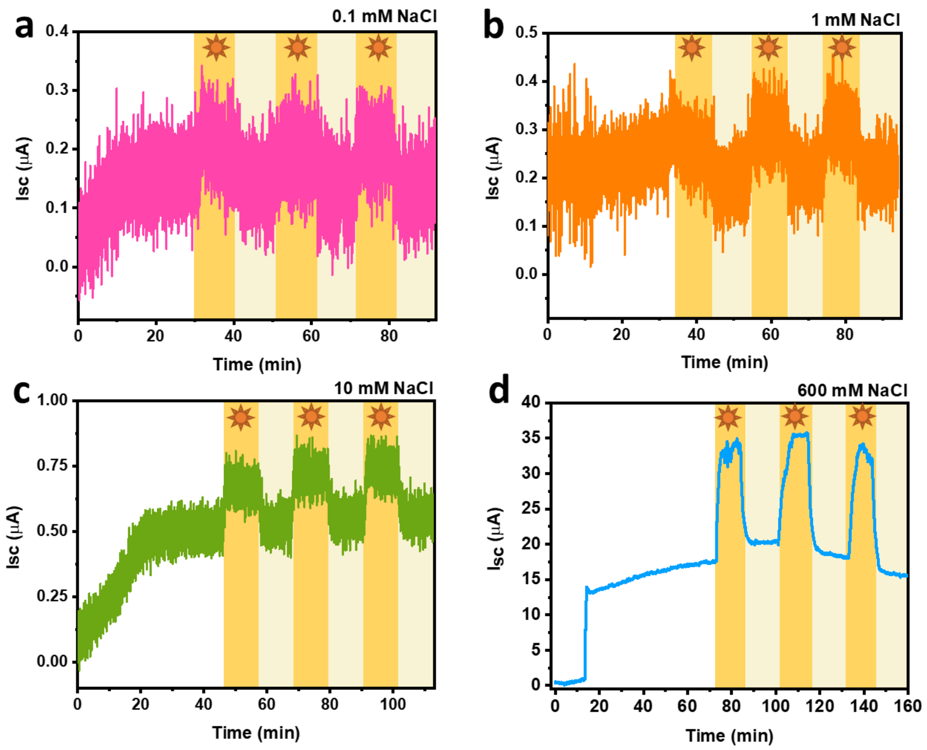

Figure S18. Measured I_{sc} of CBAP membrane immersed in 0.1mM (a), 1mM (b), 10mM (c) and 600mM (d) NaCl electrolyte solution when the solar light (1 sun illumination) was periodically turned on and turned off.

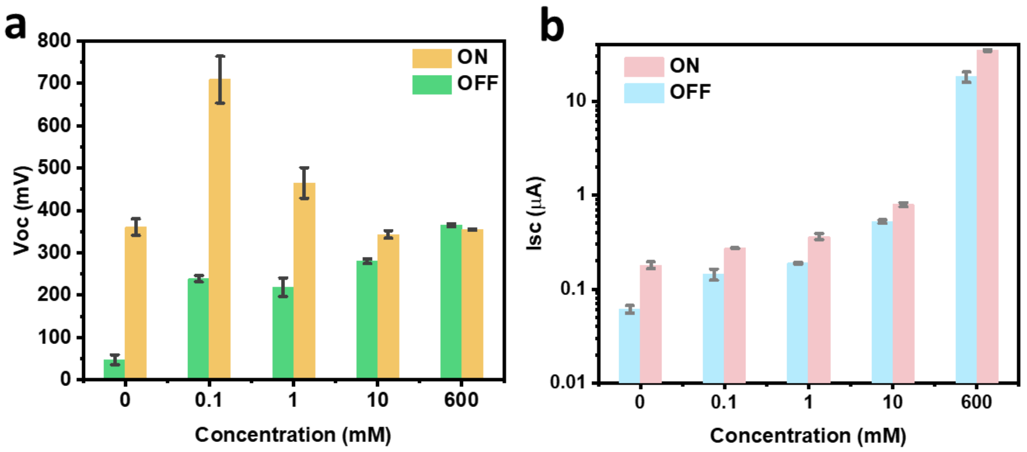

Figure S19. Measured V_{oc} (a) and I_{sc} (b) of CBAP membrane immersed in different concentration of NaCl electrolyte solution. The solar light (1 sun illumination) was turned on and turned off periodically.

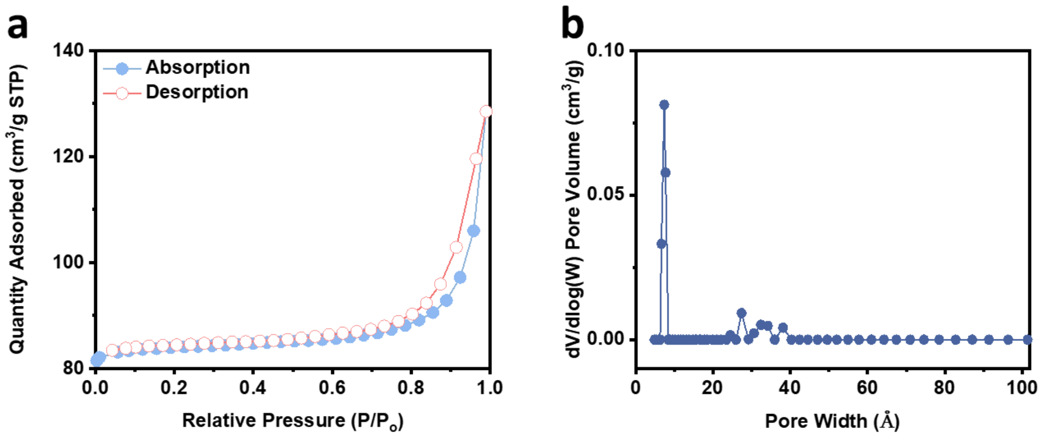

Figure S20. The BET adsorption isotherm (a) and pore size distribution (b) of CBAP membrane.

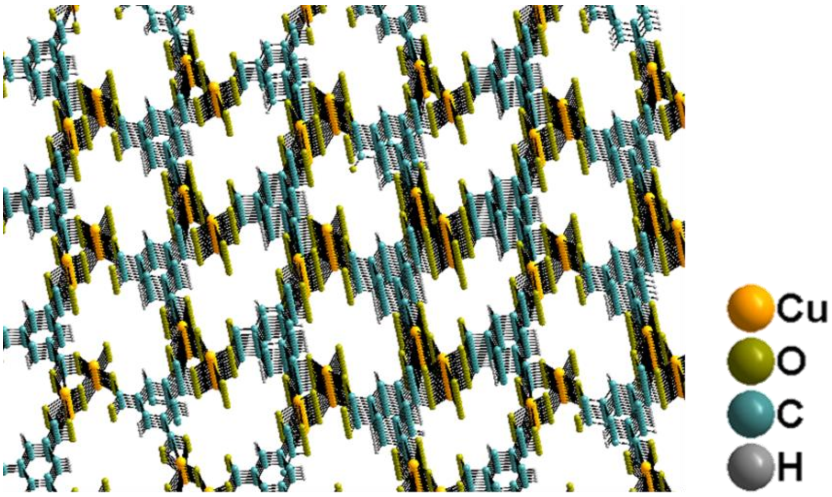

Figure S21. The crystal structure of CBA.

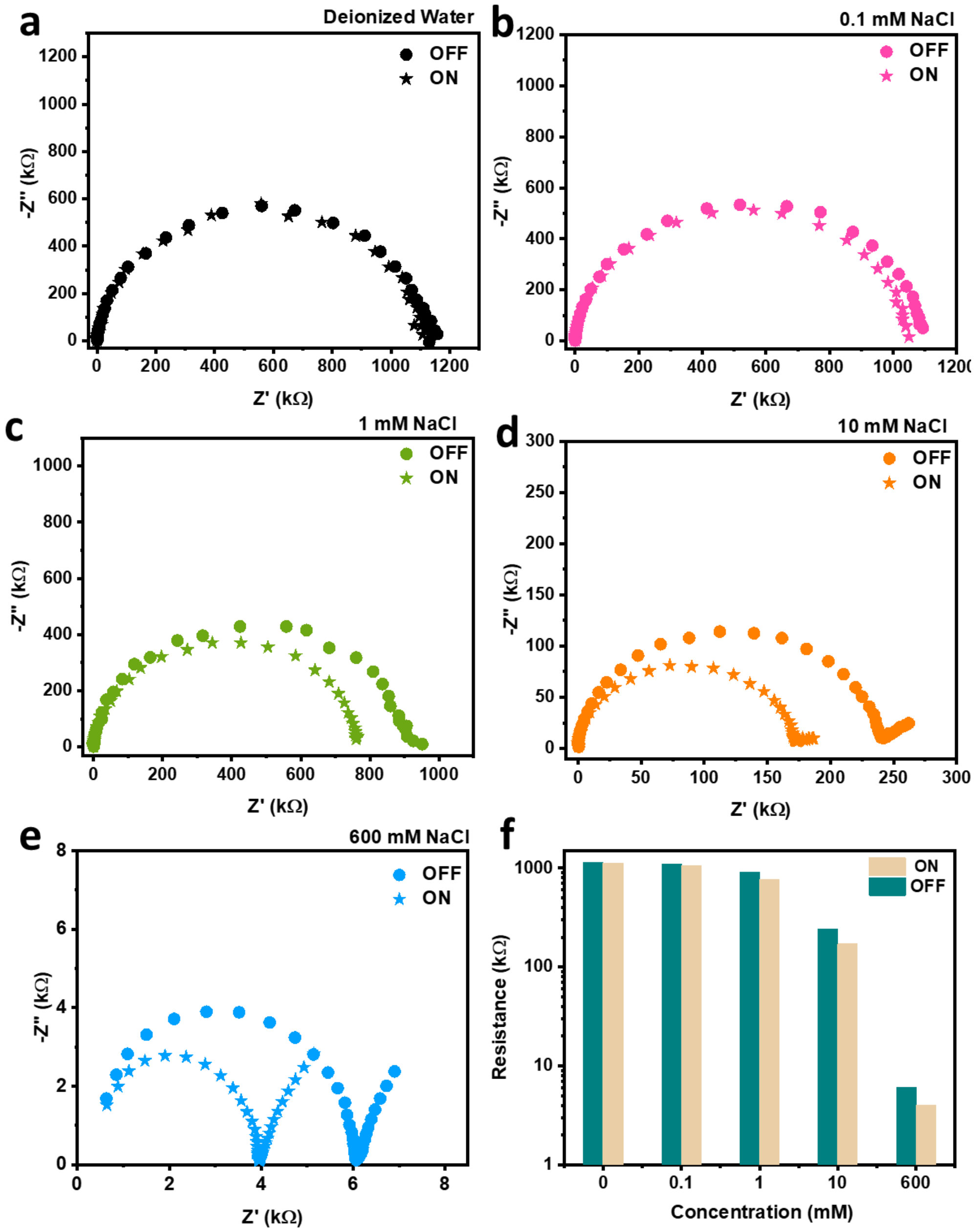

Figure S22. The impedance of CBAP membrane immersed in deionized water (a), 0.1mM (b), 1mM (c), 10mM (d), and 600mM (e) NaCl electrolyte solution. Measured impedances (f) of CBAP membranes immersed in different concentration of NaCl electrolyte solution. The solar light (1 sun illumination) was turned on and turned off periodically.

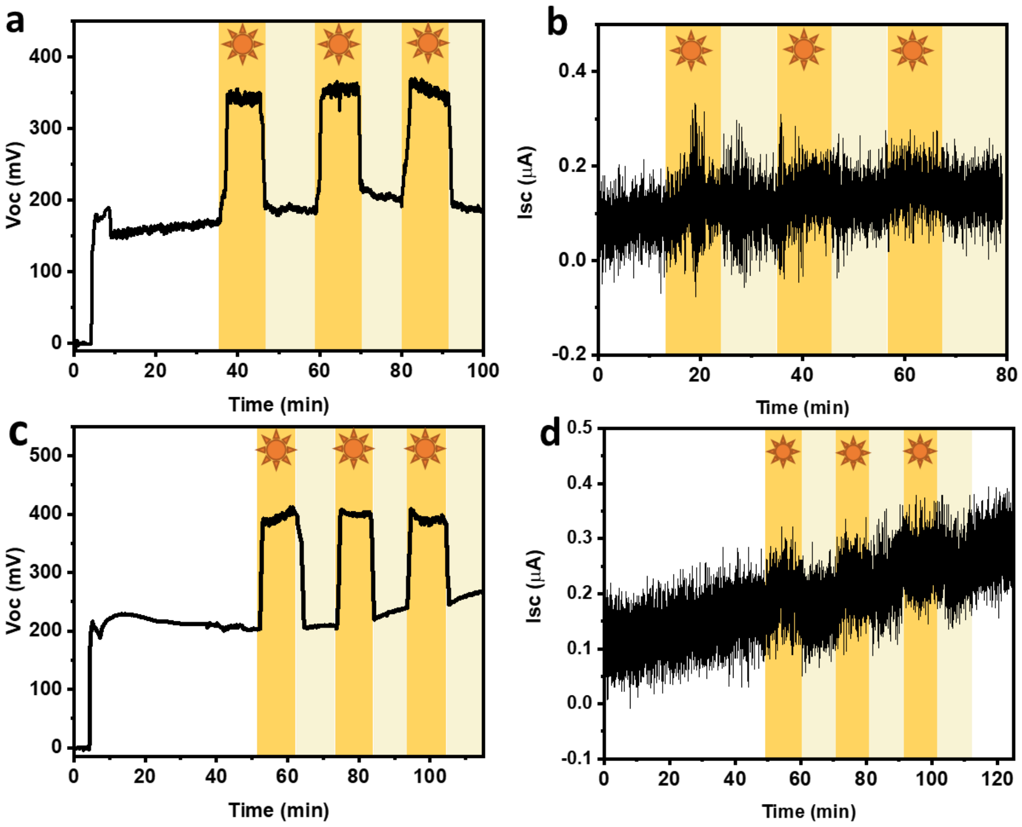

Figure S23. Measured V_{oc} (a) and I_{sc} (b) of CBAP membrane with length of 1.5 cm. Measured V_{oc} (c) and I_{sc} (d) of CBAP membrane with length of 2.5 cm. The electrolyte solution was 1 mM NaCl and the solar light (1 sun illumination) was periodically turned on and turned off.

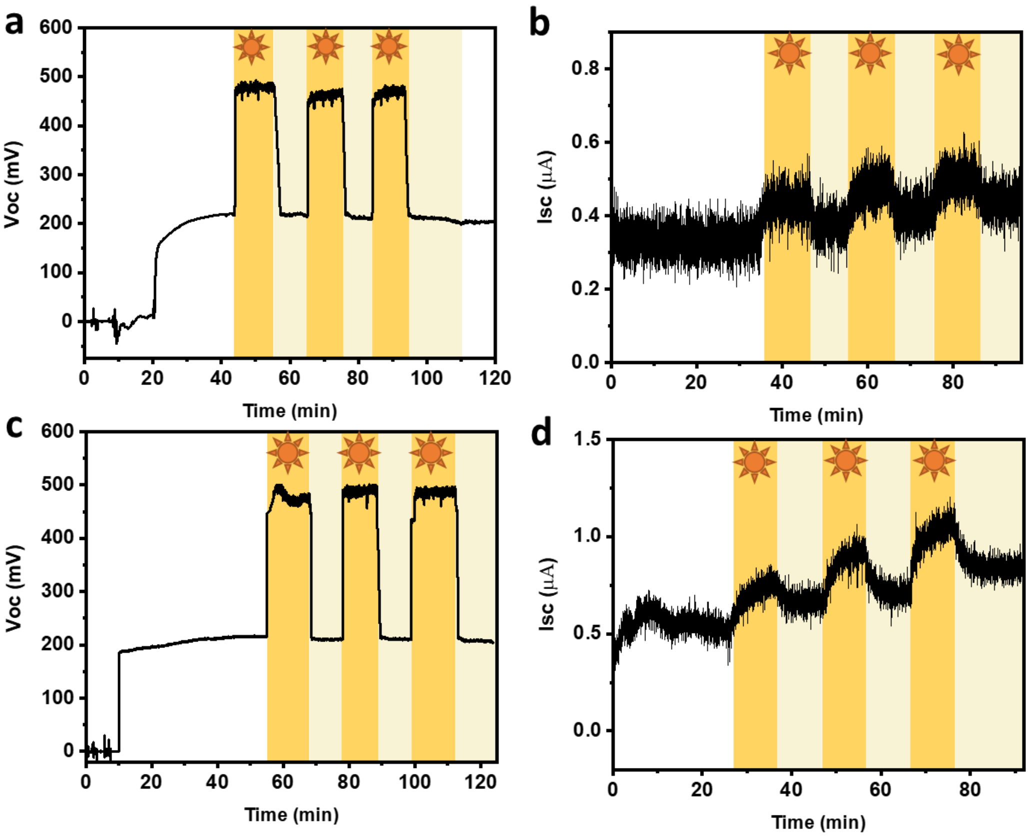

Figure S24. Measured V_{oc} (a) and I_{sc} (b) of CBAP membrane with width of 2cm. Measured V_{oc} (c) and I_{sc} (d) of CBAP membrane with width of 3 cm. The electrolyte solution was 1 mM NaCl and the solar light (1 sun illumination) was periodically turned on and turned off.

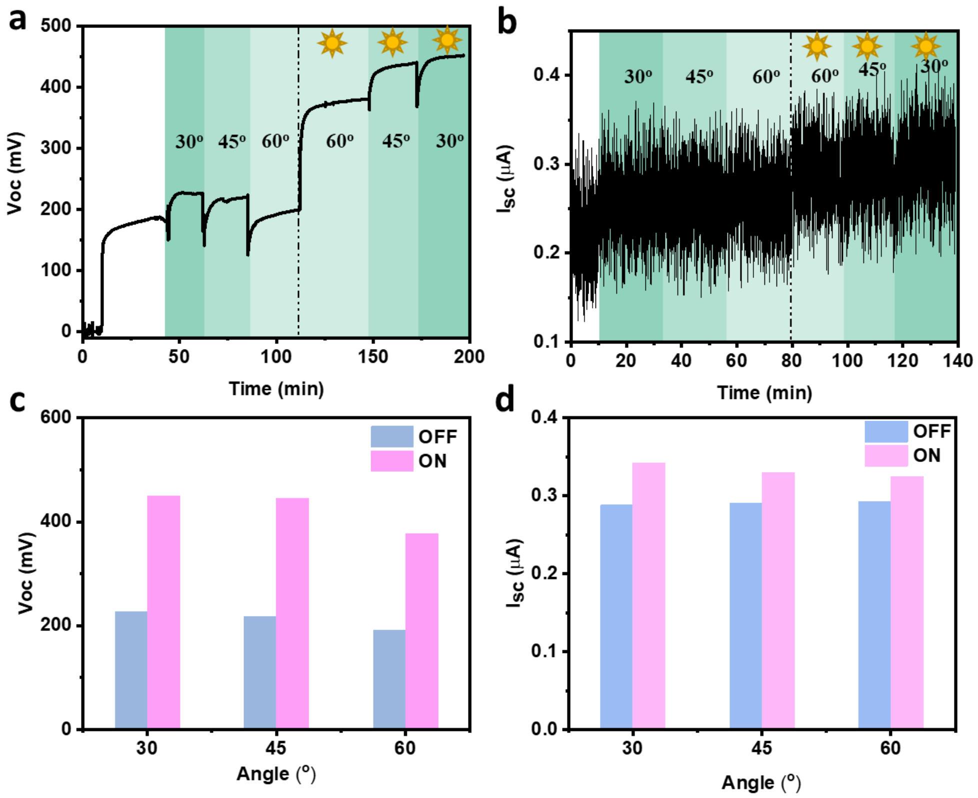

Figure S25. Measured V_{oc} (a, c) and I_{sc} (b, d) of CBAP membrane with different angles (30°, 45° and 60° respectively). The electrolyte solution was 1mM NaCl and the solar light (1 sun illumination) was periodically turned on and turned off.

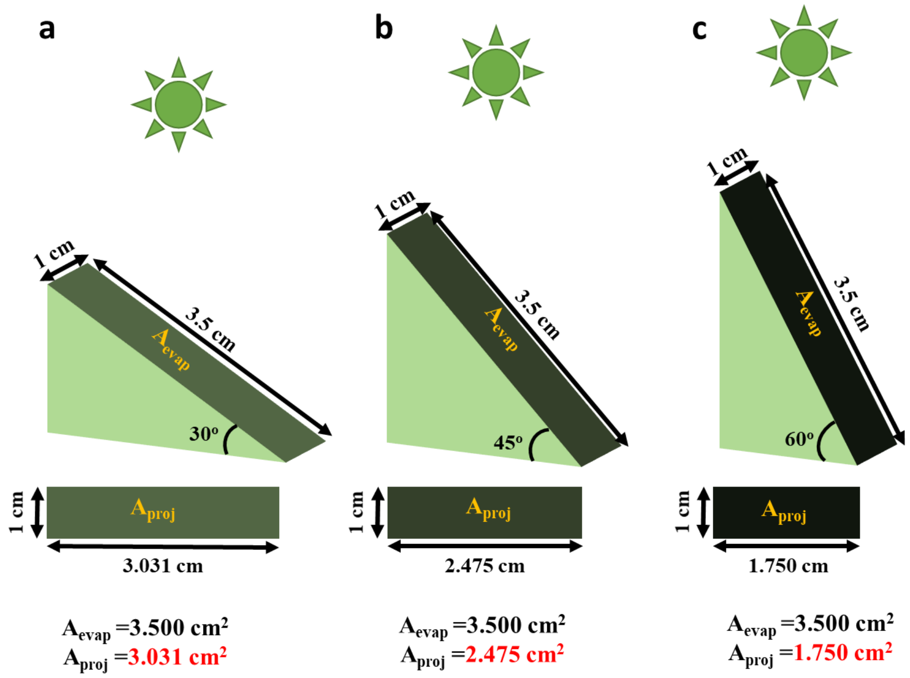

Figure S26. The evaporation area (A_{evap}) and projected area (A_{proj}) of CBAP membrane illuminated under solar light with different angles.

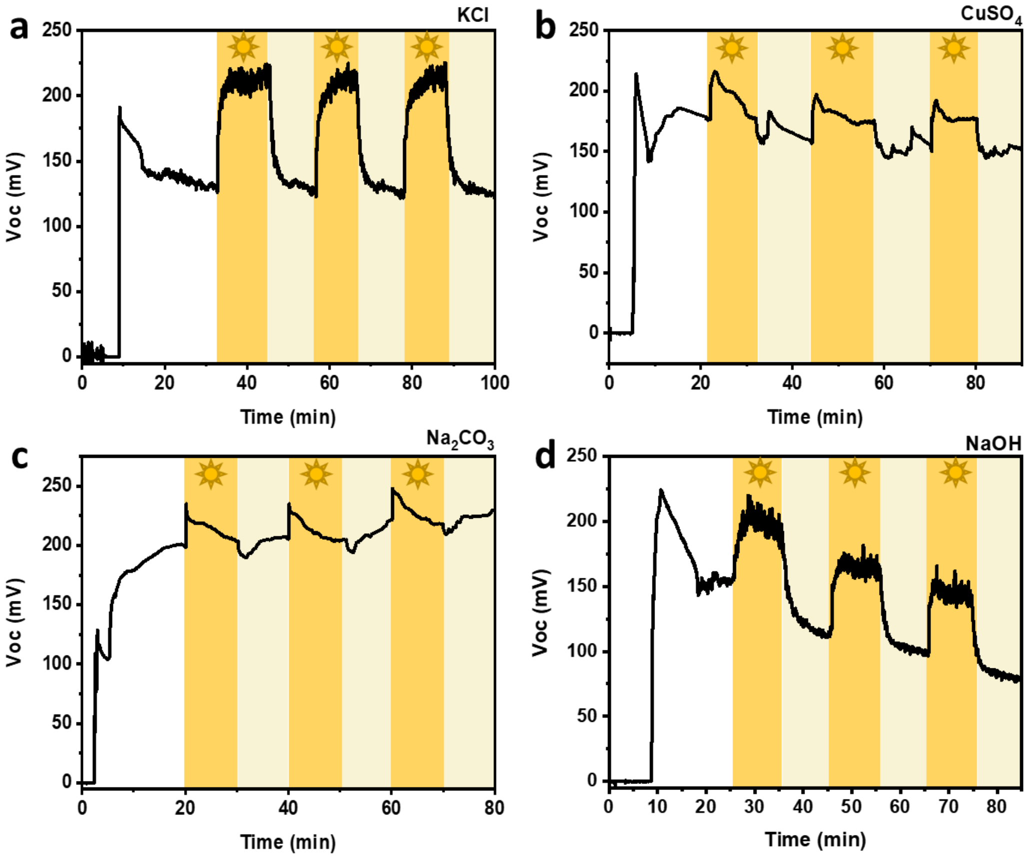

Figure S27. Measured V_{oc} of CBAP membranes immersed in 1mM KCl (a), $CuSO_4$ (b), Na_2CO_3 (c) and NaOH (d) respectively, when the solar light (1 sun illumination) was periodically turned on and turned off.

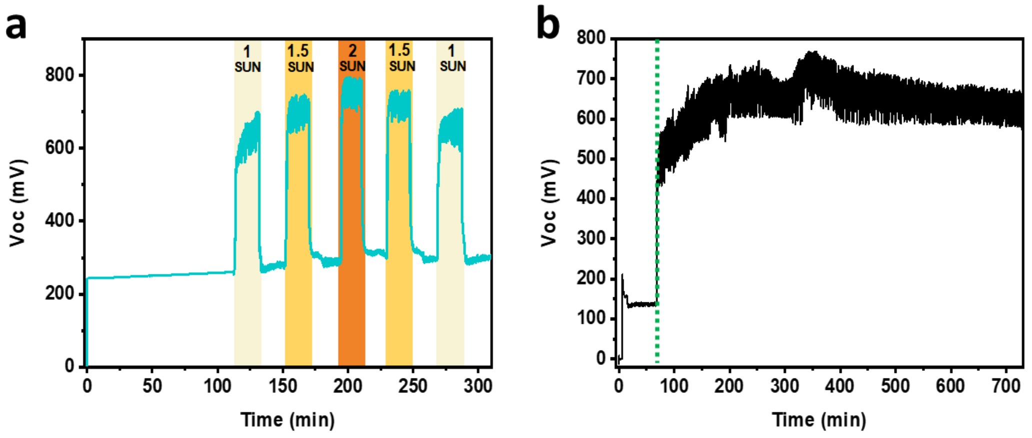

Figure S28. a) Measured V_{oc} of CBAP membrane immersed in 0.1mM NaCl electrolyte solution under different solar intensity (1, 1.5 and 2 sun illumination) when the solar light was periodically turned on and turned off. b) Sustainability of electricity generation of CBAP membrane immersed in 0.1mM NaCl electrolyte solution under 1 sun illumination.

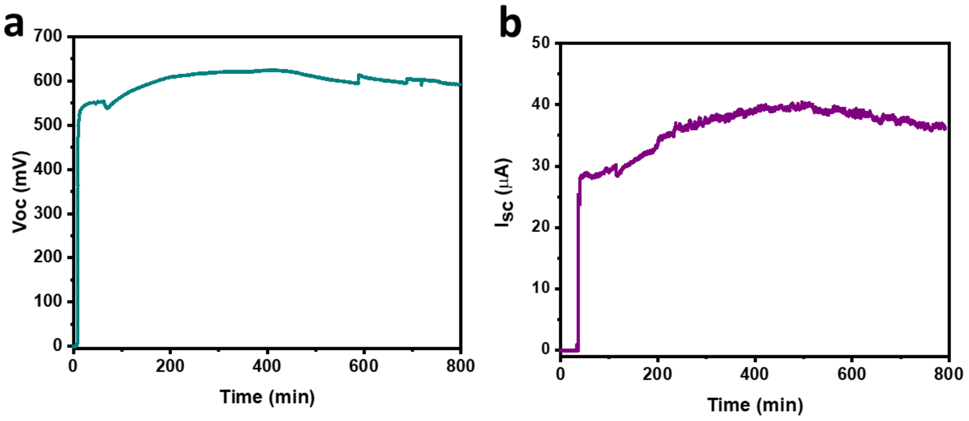

Figure S29. Sustainability of V_{oc} (a) and I_{sc} (b) of CBAP membrane in sea water desalination process under 1 sun illumination.

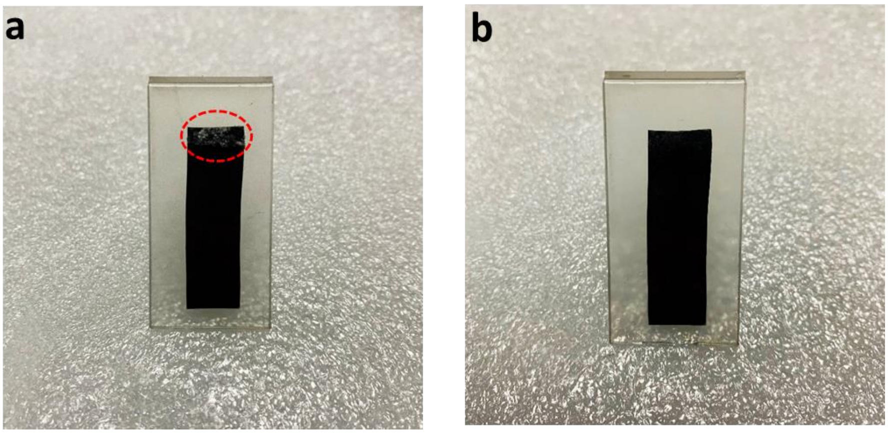

Figure S30. The optical photographs of CBAP membrane after sea water desalination for 12 hours under one sun intensity (a) and CBAP membrane after turning off solar light for 30 minutes (b).

Table S1. The Specific calculation results of evaporation rate and solar-vapor efficiency under one sun illumination.

Sample	T_0 [℃]	T [℃]	H_{sen} [J g^{-1}]	$\triangle H_{vap}$ [J g^{-1}]	h_{LV} [J g^{-1}]	Evaperation Rate [kg m^{-2} h^{-1}]	m [kg m^{-2} h^{-1}]	Solar-vapor efficiency [%]
PVDF	25.2	26.2	4.2	2440.2	2444.4	0.4523	0.3323	22.6
CBA	25.1	33.6	35.7	2425.3	2461.0	1.0014	0.8814	60.3
CBAP	25.1	38.4	55.9	2416.0	2471.9	1.4424	1.3224	90.8

Table S2. The Debye length in different concentrations of NaCl solution.

Concentration[mM]	10^{-7}	0.1	1	10	600
Debye length [nm]	961.33	30.40	9.61	3.04	0.39

Table S3. The impedance of CBAP membrane immersed in different concentrations of NaCl electrolyte solution when the solar light (1 sun illumination) was turned on and turned off.

NaCl [mM]	0	0.1	1	10	600
V_{OFF} [mV]	47.374	239.179	218.700	280.079	638.790
V_{ON} [mV]	360.674	709.260	465.207	343.460	620.147
I_{OFF} [μA]	0.061	0.144	0.189	0.524	18.181
I_{ON} [μA]	0.180	0.273	0.362	0.792	34.715
R_{OFF} [kΩ]	1141.333	1092.200	910.500	242.000	6.090
R_{ON} [kΩ]	1122.587	1063.500	769.533	170.933	3.989
ΔV [mV]	+313.299	+470.081	+246.507	+63.381	-18.643
ΔR [kΩ]	-18.746	-28.700	-140.967	-71.067	-2.101
$\Delta V / V_{OFF}$ [%]	+661.327	+196.539	+112.715	22.630	-2.918
$\Delta R / R_{OFF}$ [%]	-1.642	-2.628	-15.482	-29.366	-34.499
$P_{max\text{-}on}$ [mW m^{-2}]	0.046	0.138	0.120	0.194	15.377

Table S4. The performance of CBAP membranes with different sizes and angles immersed in 1mM NaCl electrolyte solution when the solar light (1 sun illumination).

Sample	Length (cm)	Width (cm)	Angle (°)	V_{OFF} (mV)	V_{ON} (mV)	I_{OFF} (μA)	I_{ON} (μA)	P_{OFF} (nW)	P_{ON} (nW)	$P_{max-off}$ (mW m^{-2})	P_{max-on} (mW m^{-2})
1	1.5	1	45	188.32	357.47	0.122	0.188	22.975	67.204	0.0383	0.1120
2	2.5	1	45	208.72	399.78	0.145	0.265	30.264	105.942	0.0303	0.1059
3	3.5	1	45	218.70	465.21	0.189	0.362	41.334	168.405	0.0295	0.1203
4	3.5	2	45	218.03	461.81	0.407	0.543	88.738	250.763	0.0317	0.0896
5	3.5	3	45	209.00	493.38	0.526	0.745	109.934	367.568	0.0262	0.0875
6	3.5	1	30	226.12	449.53	0.288	0.342	65.123	153.739	0.0465	0.1098
7	3.5	1	45	217.51	445.34	0.290	0.330	63.078	146.962	0.0451	0.1050
8	3.5	1	60	190.87	376.46	0.292	0.324	55.734	121.973	0.0398	0.0871

Table S5. An up-to-date summary of materials used for evaporation-driven electricity generation.

Solar absorber materials	Evaporation-driven electricity	Interfacial solar evaporation	Sea water desalination	Solar intensity [kW m^{-2}]	Electrolyte solution	Evaporation rate [kg $m^{-2}h^{-1}$]	Solar-vapor efficiency [%]	Voltage [V]	Power density [mW m^{-2}]	Ref.
Carbon film	√	×	×	/	Water	/	/	1.0	0.126	[40]
Carbon black	√	√	×	1	Water	/	85%	0.005	Unknown[a]	[15]
Carbon black	√	×	×	/	Water	/	/	1.0	0.212	[8]
Natural wood	√	×	×	/	Water	/	/	0.3	1.2	[19]
Silicon nanowire	√	×	×	/	Water	/	/	0.4	60	[3]
Fabric pieces	√	×	×	/	1mM NaCl	/	/	0.7	0.031	[18]
UIO-66/AlOOH	√	×	×	/	Water	/	/	1.63	0.150	[12]
Graphene/carbon cloth	√	√	×	1	0.5 M NaCl	1.3	83	0.37	Unknown[b]	[14]
Carbon nanotubes /cellulose paper	√	√	√	1	0.6 M NaCl	1.15	/	0.6	Unknown[c]	[16]
GO/cellulose ester film	√	√	√	1	0.6 M NaCl	1.30	86	0.31	Unknown[d]	[13]
Hybrid MOFs	√	√	√	1	0.6 M NaCl	1.874	90.8	0.620	15.377[e]	This work

[a,b)] The maximum area output power density was not mentioned in the work. c) The maximum output power of carbon nanotubes /cellulose paper is 2.1μW, but its maximum area output power density was unable to calculate because its size was not given. [c)] The maximum area output power density was unable to calculate because the thickness of graphene oxide/mixed cellulose ester film was not given but its maximum bulk output power density was 20.5 μW cm^{-3}. [e)] In our study, the size of a single hybrid membrane with PVDF substrate is about 1cm×3.5cm×100μm, so the corresponding maximum bulk output power density of a single membrane is 26.78 μW cm^{-3}, which was higher than that of the graphene oxide/mixed cellulose ester film.
